# CardioRespiratory Effects of Wildfire Suppression (CREWS) study: an experimental overview

**DOI:** 10.3389/fpubh.2025.1578582

**Published:** 2025-05-16

**Authors:** L. Madden Brewster, Drew Lichty, Natasha Broznitsky, Philip N. Ainslie

**Affiliations:** ^1^Centre for Heart, Lung and Vascular Health, School of Health and Exercise Sciences, University of British Columbia, Kelowna, BC, Canada; ^2^Canada Wildfire, University of Alberta, Edmonton, AB, Canada; ^3^Research and Innovation Business Area, British Columbia Wildfire Service, Victoria, BC, Canada

**Keywords:** cardiorespiratory, wildfire, occupational and environmental exposure, occupational health, wildland firefighters

## Abstract

An increase in the severity of global wildfires necessitates examination of the associated health risks, particularly in wildfire personnel. Exposure to particulate matter from smoke (PM_2.5_), soil/dust, and ash (PM_4_) and other wildfire-associated pollutants (carbon monoxide) have previously been linked to acute cardiovascular and respiratory dysfunction. Despite mounting epidemiological evidence of cardiorespiratory-related morbidity and mortality related to wildfire suppression exposures, the chronic effects (>1 year) of wildland firefighting on the pathophysiological progression of cardiorespiratory disease in this vulnerable group remain largely uncharacterized. Thus, a repeated-measures study with open recruitment over 3-years was designed in partnership with the University of British Columbia Okanagan and the British Columbia Wildfire Service (BCWS) to address gaps in wildland firefighter (WFF) health research. The primary aims of the CardioRespiratory Effects of Wildfire Suppression (CREWS) Study are to: 1) Examine the chronic effect(s) of wildfire suppression on selected aspects of vascular and respiratory health in BCWS WFFs, 2) Examine cardiorespiratory effect(s) of acute (e.g., cross-shift) wildfire suppression, and 3) Identify mechanisms contributing to the progression of wildfire-associated cardiorespiratory dysfunction in WFFs. To address these aims, as detailed in this overview, selected clinical and subclinical cardiorespiratory measures, circulating and airway-specific inflammatory biomarkers, heavy metal exposure, and personal air sampling methods to detect wildfire smoke, dust, and ash exposure will be employed across three consecutive wildfire seasons in the same cohort of BCWS WFFs. The findings from this study will provide new insight into the short and long-term impact of wildland firefighting on cardiorespiratory health. This information will inform guidelines and development of future mitigation strategies to improve long-term health and safety in WFFs.

## Introduction

Worldwide, firefighting personnel are employed seasonally to combat wildland fires, typically during periods when risks are highest (1, 2). Climate change has exacerbated the occurrence and severity of wildfire seasons in Canada, including British Columbia (BC), over the past 50 years (3). In 2023 alone, more than 15 million hectares of land burned across Canada, twice that of the previous 1989 record (4). With fire seasons becoming progressively hotter, drier and lengthier, along with the widening of the wildland-urban interface, wildfire suppression efforts to protect human life, resources, property, and other assets are growing more complex, high-risk, and necessary. In BC, wildfire management is led by the BC Wildfire Service (BCWS) who routinely employ nearly 1,300 wildland firefighters (WFFs) every year—although these numbers can markedly swell in response to the severity of the season (5). With limited seasonal turnover rates, nearly 1,000 BCWS WFFs are employed across consecutive seasons (6). In addition to BCWS employees (type 1 WFFs), thousands of contracted groundcrew members (type 2 and 3 WFFs) also aid in suppression efforts across BC each fire season, increasing the size of this vulnerable worker cohort (7).

Similar to other regions, Canadian WFFs work long hours in dangerous and often remote environments; they are exposed to a complex mixture of hazards including chemical (smoke, ash, dust, engine exhaust), physical (heat, noise, hand-arm vibration, UV radiation, danger trees), and psychosocial hazards (extended work hours, shiftwork, fatigue, mental health consequences) (8). A typical WFF crew deployment consists of 12 to 16-hour shifts for up to 14 consecutive duty-days (>7 h shift) on the fire line (i.e., the location where ground crews engage in wildfire suppression), with 2–6 days of rest in-between deployments (9). On the fire line, a crew's task is either to 1) contain the fire's edge by removing adjacent forest fuels using chainsaws and hand tools and to suppress any remaining fire activity within a prescribed distance from the fire perimeter (direct attack) or, 2) establish a containment line some distance from the fire perimeter to allow the fire to burn toward or conduct controlled ignitions to bring the fire's edge to the newly established perimeter (indirect attack) (8). Direct suppression is carried out via water delivery from hoses (supplied by high-pressure water pumps) as well as using hand tools to disperse the hot ash and coals (8). Due to their work directly at or near the fire perimeter, WFFs are often exposed to ambient wildfire smoke that broadly impacts the local air quality, as well as direct smoke due to working adjacent to and upwind of smoldering and flaming hotspots (10). The manipulation of ash, dust, and coals to suppress fire behavior also liberates significant particulate into the air and within the breathing zone of the WFF, as well as deposits smoke, ash, and soot directly onto the skin (10–12). Unlike structural or urban firefighters, WFFs do not use a self-contained breathing apparatus during fire suppression and often lack other respiratory protective equipment (RPE) altogether, thereby increasing exposure and limiting the applicability of urban firefighter health research to WFFs specifically (8). During a deployment, it is common for WFFs to spend non-working hours at open-air fire camps situated near the fire of interest, posing additional risk of exposure to smoke, dust, heat, and noise during recovery hours (13). Generally, the wildland fire line is a hazardous work environment that is difficult to control due to the remote environments, dangerous terrain, and dynamic conditions of weather and fire behaviors (8).

The occupational exposures of wildland firefighting primarily comprise a mixture of vegetative smoke, dust/soil (including crystalline silica) and ash particulates which emanate from ambient air quality conditions as well as task-specific disturbances that increase exposures. These particulates range in size depending on their origin whereby fine particulate matter (PM_2.5_; aerodynamic diameter <2.5 μm) largely represents wildfire smoke and respirable particulate matter (PM_4_; aerodynamic diameter <4 μm) primarily reflects particulates from soil/dust and ash (10). PM_2.5 − 4_ can travel deep into the airways to directly disturb the respiratory tissues; and, in the case of PM_2.5_, can cross the alveolar-capillary membrane to further damage the vasculature (14, 15), potentially impairing gas exchange. This may be particularly true for wildfire smoke PM which is overwhelmingly present in the submicron size fraction (< PM_1_) (16, 17). Additional components of wildfire smoke include acrolein, benzene, formaldehyde, carbon dioxide, carbon monoxide, nitrous oxides, sulfur dioxide, and carcinogenic polycyclic aromatic hydrocarbons, which, when studied in isolation result in health complication; in combination these components may exacerbate risk (18).

Mounting evidence suggests that these exposures are not without significant detriment to WFF health (19, 20). Namely, wildfire smoke poses an acute vascular and respiratory hazard and is associated with cardiorespiratory-related hospitalizations and mortality in the general population (21–29). In WFFs, Navarro et al. estimated the relative risk of lung cancer and cardiovascular disease mortality using established PM_2.5_ exposure-response relationships by substituting PM_4_ data collected on fire lines and found an increased risk of cardiovascular disease (16–30%) and lung cancer (8–43%) dependent upon career (5–25 years) and season length (48 and 95 days) (30). Recent work compiled from Alberta Wildfire employment records with linked administrative health and cancer records in 12,731 WFFs from 1998 to 2022 identified an increased risk of chronic obstructive pulmonary disease, pneumonia, asthma, and cardiovascular disease, but not cancer risk, proportional to cumulative hours of sustained attack (e.g., ground crew personnel) wildland firefighting after adjusting for appropriate covariates (age, sex, and inferred First Nations origin) (31). Acutely, clinical indicators of lung dysfunction such as forced expiratory volume in 1 second (FEV_1_) and the ratio of FEV_1_ to forced vital capacity (FVC) have been shown to be reduced cross-shift and cross-season in WFFs (32–35). These declines in lung function, based on high exposure, may also be paired with subsequent clinical diagnosis or morphological changes such as the onset of asthma, bronchial wall thickening, and reduced diffusion capacity of the lungs in the aftermath of responding to large fire events (36). The mechanisms for exposure-induced respiratory changes are not entirely clear, although increases in circulating pro-inflammatory markers, such as interleukin (IL)-8, C-reactive protein, and serum amyloid A, have been observed cross-shift with evidence of these changes associated with burn-specific tasks (e.g., drip torch use) compared with fire perimeter management (e.g., holding) (37). In addition, upper and lower respiratory symptoms in wildland firefighters are associated with increased markers of airway inflammation (eosinophilic cationic protein and myeloperoxidase) in sputum and nasal lavage fluid from pre- to post-season (38). Overall, data indicating respiratory insult in wildfire personnel are mounting, but long-term data with mechanistic details remain limited.

The vasculature may also be compromised by the occupational exposures of wildfire suppression such as smoke exposure. For example, in a double-blind crossover study, healthy adults exposed to 3-h of woodsmoke (mean particle concentration 314 μg/m^3^) whilst undergoing intermittent exercise, had increased central arterial stiffness, compared with filtered air (39). On the contrary, vascular dysfunction determined by pulse wave velocity (arterial stiffness) and vasomotor function was unchanged immediately after, and fibrinolytic capacity was unchanged 2-h after intermittent exercise in a higher concentration of particulate (1 mg/m^3^) for 1-h (40). In WFFs, maximal oxygen consumption decreased 4.1 mL/kg/min from pre- to post-season with those reporting >640 h of hazard pay having a significantly greater reduction (−7.1 mL/kg/min) than those with less hazard pay (−1.7 mL/kg/min) (41). Additionally, a negative correlation (r=0.-47) was found between hazard pay and ankle-brachial index, indicating an association between increased exposure to the wildfire environment and diminished peripheral vascular health in WFFs (41). Recent evidence in 1,051 WFFs aged 17 to 64 years demonstrates a 2.84 fold greater odds of developing hypertension and prehypertension than the general U.S. population, a primary risk factor for cardiovascular disease (42). Cross-season increases in total body, fat, and visceral fat mass as well as total cholesterol, low density lipoprotein, and total globulin have been observed in a group (*n* = 27) of Alaskan WFFs, metabolic changes consistent with chronic inflammation and cardiovascular risk (43). Wildfire smoke indeed induces inflammatory and oxidative burden, known contributors to cardiovascular risk (30, 44–46). Although a growing body of research supports that the occupational exposures associated with wildland firefighting contribute to poor cardiorespiratory health outcomes, a large gap remains in identifying the etiological progression of such disparities and the potential cumulative effect of repeated bouts of occupational exposure to wildfire smoke, dust and ash.

To address these gaps, in January 2023, the University of British Columbia Okanagan (Kelowna, BC, Canada) approached the BCWS Research and Innovation Business Area (R&I) with a proposal examining the chronic (>1 year) cardiorespiratory health effects of consecutive seasons of wildland firefighting. This essential partnership formed the basis for the co-development of a feasible, repeated measures study with open enrollment of BCWS WFFs across 3-wildfire seasons (2024–2026) termed the CardioRespiratory Effects of Wildfire Suppression (CREWS) Study. The primary aims of the CREWS Study are to: 1) Examine the chronic effect(s) of wildfire suppression on selected aspects of vascular and respiratory health, 2) Examine cardiorespiratory effect(s) of acute (i.e., cross-shift) wildfire suppression, and 3) Identify mechanisms contributing to the progression of wildfire-associated cardiorespiratory dysfunction in BCWS WFFs. The Wildland Firefighter Exposure and Health Effects (WFFEHE) was a seminal study conducted in 2018–2019 by the Center for Disease Control's National Institute for Occupational Safety and Health, United States Forest Service, and Department of the Interior that addressed similar aims regarding heart, lung, kidney, and hearing health of WFFs (47). Unfortunately, this study was prematurely halted after 2-years due to the coronavirus disease 2019 pandemic. The CREWS Study complements and extends the aims of the WFFEHE study by; 1) increasing the study length; 2) providing comprehensive assessment of cardiorespiratory health using gold standard and subclinical techniques; and 3) implementing novel research aims to comprehensively identify cardiorespiratory function in WFFs based on emerging health research and relevant findings. Herein, we outline the experimental design, methods, considerations, strengths, and limitations of the CREWS Study following completion of the first year of data collection.

## Methods

### Ethical approval

This study was approved by the University of British Columbia Clinical Research Ethics Board (H23-02942) in accordance with the Declaration of Helsinki, except for registration in a database. All participants received thorough explanation of the study and provided written, informed consent prior to testing. Notably, researchers emphasized that participation was independent of—and would not influence—an individual's standing within the BCWS. All participants were free to withdraw without justification or penalty from experimentation at any time. Participants were also informed that they could opt out of any individual portion of the study (e.g., venous blood draw) if preferred. Participants were tested during working hours and therefore compensated according to their hourly wage, inclusive of over-time pay when applicable.

### Participants

The research team worked with BCWS R&I to identify suitable WFF crews based on proximity to the research team, anticipated crew seasonal access, expected crew member retention rate, previous familiarity with research, and personal crew interest. Thus, ground crews (unit and initial attack crews) from two BCWS fire bases within the Coastal Fire Center, were identified as the recruitment pool, with one base as the primary recruitment site for the study. Potential recruits included members of the BCWS from two unit crews and five initial attack crews who met inclusion criteria. The inclusion criteria were: 1) >17 years of age, 2) meets health standards outlined in physician release form required of type 1 firefighters in Canada to complete mandatory fitness testing (48), 3) wildfire ground crew personnel with seasonal BCWS contracts, and 4) available for post season data collection. The research team conducted participant recruitment across two group information sessions conducted in April and May of 2024, 1 day prior to a testing window. During each of these recruitment sessions, the research team explained the study in its entirety and offered the opportunity for questions. Thereafter, individuals could sign up to participate in the subsequent testing days based on individual and crew schedules. An open enrollment is adopted throughout the study—into 2026—to account for changes in crew retention and to promote a diverse demographic, inclusive of diverse firefighting experience, amongst the participants. Similar pre-season recruitment sessions for new recruits will be provided for future seasons. Although a major reason these crews were chosen was due to higher retention rates compared with other crews within the organization, an open enrollment will allow for increased new recruits (1^st^ year WFFs) and female WFF enrollment, which may otherwise be limited by a single enrollment period. An example of the current enrollment from the 2024 season is provided in Table 1. In total, 52 participants enrolled in the study across the 2024 pre-season testing windows. An additional 4 participants who were not present for pre-season testing enrolled during the post-season testing windows, so the current total enrollment of the study is 56 participants. Only 5 enrolled participants from these are female. Forty-eight individuals from the pre-season testing were also tested in the post-season, 1 of which were female. The primary reasons for attrition were: 1) participant finished prior to seasonal contract end (using earned time off) 2) participant was no longer interested in participating without specific reason.

**Table 1:** Enrolled participants across 2024 season^*^.

**Crew**	**Pre-season**		**Post-season**	
	**Males**	**Females**	**Males**	**Females**
UC #1	17	0	14	0
UC #2	19	1	19 (2)	1
IACs	13	2	11	1 (2)
Total	49	3	44 (2)	2 (2)

Numbers in parentheses show newly enrolled participants as per the open enrollment of the CREWS Study. In total, 56 wildland firefighters are enrolled with 46 paired samples in the 2024 season. UC, unit crew; IACs, initial attack crews. ^*^Open enrollment will continue in 2025 and 2026.

### Sample size calculations

Sample size calculations vary based on the outcome measured and timepoint assessed (e.g., longitudinal vs. acute studies). For most of the outcomes (oscillometry, gas exchange, sputum and serum markers, FMD, PWV) in the CREWS Study, previous data in WFFs is not available or not appropriately reported (e.g., means and standard deviations) to determine average effect sizes cross-shift and seasonally. However, we averaged the Cohen's D based on cross-seasonal changes in FEV_1_ in WFFs previously reported (32, 49) to estimate an effect size of 0.58 for longitudinal respiratory outcomes. Acute changes in FEV_1_ (38, 49) are similarly shown to produce an effect size 0.51. Thus, sample size calculations for the longitudinal study were performed in G^*^power software using 0.58, setting α to 0.05 with a minimum power of 0.8. As the software does not have the option to run mixed models, repeated measures analysis of variance was used with the number of groups set to 2 to account for years of firefighting experience (novice vs. experienced) and 6 timepoints (number of measurements). A total sample size of 26 was estimated to be needed for the longitudinal study. The acute studies sample size—also 26—was determined by dependent one-tailed *t*-tests, an effect size of 0.51, setting α to 0.05 with a minimum power of 0.8. To account for up to 25% dropout based on average BCWS turnover rates and other factors, this sample size for the longitudinal study was increased to 33. However, any individual from the recruitment pool was enrolled if they were interested due to the value of personal health data gained from participation in the study. Moreover, to account for demographic comparison, as well as some of the added exploratory measures (see discussion), an open recruitment is planned for 2025–2026 to maximize the sample size.

### Equipment logistics

To make testing logistically feasible for participants, laboratory equipment from the University of British Columbia Okanagan (Kelowna, BC, Canada) was transported to one of the Coastal Fire Center bases central to participants (~5 h by car) to conduct all testing. As such, research equipment, infrastructure needs (e.g., bed, mattress, tables, etc.), and consumables (e.g., sterile needles, microtubes, mouth pieces, etc.) were packed in pelican cases, duffel bags, and boxes for transport to the fire base for each round of testing. Two personal SUVs were used to transport equipment. Special consideration was taken to maintain biological sample integrity during transport, which included a dewar filled with liquid nitrogen for human plasma and serum samples as well as a −20°C portable cooler (CF55, Alpicool, Guangdong, China) for sputum samples. Cooler temperature was maintained during transport via a 1,260 Wh 50.4 V portable battery (EF3 Pro, EcoFlow, Shenzhen, China). For each testing period, the laboratory was set up inside the fire base with access to continuous power and stable temperature. Equipment during research conducted on or near the fire line (e.g., staging, an anchor-point near the fire line where crews typically gather to brief/debrief about the day), such as during midseason data collection, was powered and/or recharged in the field using the same portable battery described above.

### Experimental overview

**Figure 1:**
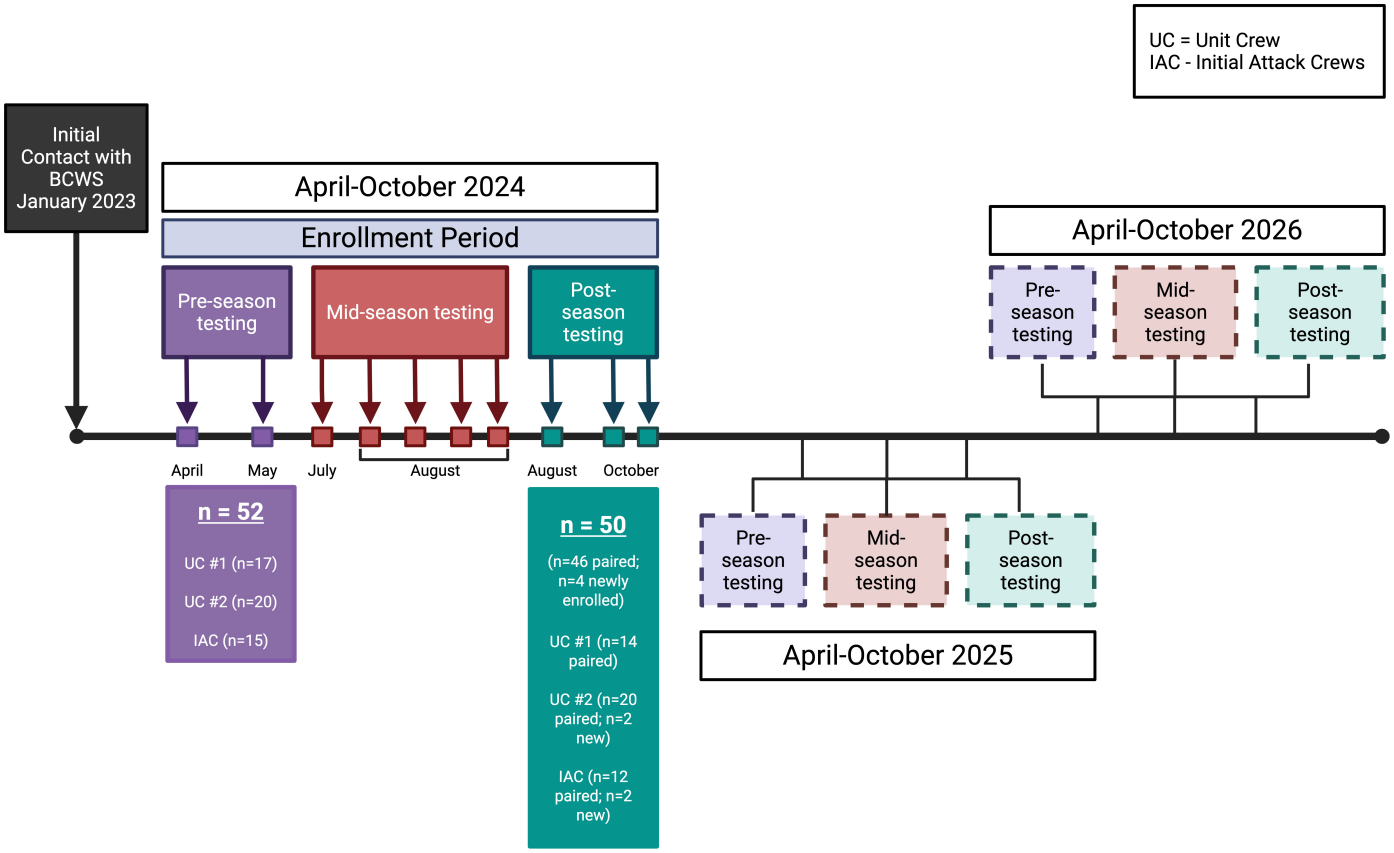
Timeline of the CardioRespiratory Effects of Wildfire Suppression (CREWS) study. Completed phases are indicated by saturated boxes, forthcoming phases are indicated by transparent boxes. Each point on the horizontal line indicates a single round of testing conducted during that phase. BCWS, British Columbia Wildfire Service.

This study is a 3-year repeated measures cohort study with open recruitment from 2024 to 2026. A primary study aim is to investigate the chronic effect(s) of wildfire suppression on selected aspects of vascular and respiratory health in BCWS WFFs, herein referred to as “longitudinal study.” Another aim is to identify and address gaps regarding acute changes in cardiorespiratory health in WFFs, conducted in the midseason, herein referred to as “acute studies.” A schematic representing the experimental overview is shown in Figure 1. Detailed methods are provided in Supplementary materials 1–3 with a table of associated materials and equipment (Supplementary Table 1).

### Experimental overview—Longitudinal study

The longitudinal study involves pre- and post-season timepoints, centering around the beginning and end of each fire season, as close to each participant's specific start and end-date as possible. The seasonal start and end of the fire season varies based on fire activity and resource needs, with seasonal contracts varying at the individual level as well. However, most seasonal contracts (85% of BCWS are seasonal contracts) begin between April and May, and in the present study is considered “pre-season.” The termination of seasonal contracts also varies, but typically occurs between August and November, considered herein as “post-season.” Both pre- and post-season testing were conducted across multiple visits by the research team, coinciding with contract start and end-dates for majority of participants. The order of participants during initial testing was determined by self-selected participant and crew availability.

**Figure 2:**
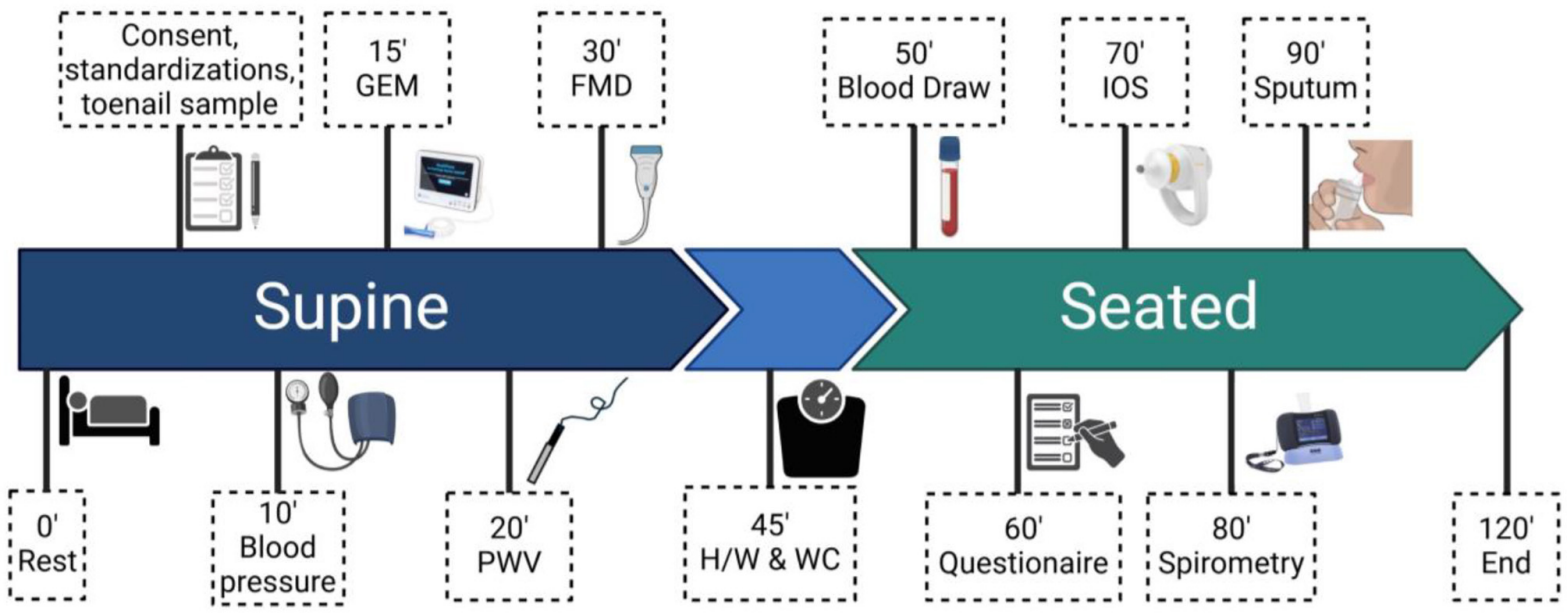
Schematic of the longitudinal study protocol conducted for pre- and post-season testing. BP, blood pressure; GEM, gas exchange monitor; PWV, pulse wave velocity; FMD, flow mediated dilation; H/W and WC, height, weight, and waist circumference; IOS, impulse oscillometry.

Participants were asked to adhere to standardizations prior to their pre- and post-season testing days. These standardizations included: fasted within 2-h, cessation of alcohol, exercise and nicotine 8-h prior, avoidance of a daily bronchodilator within 12 h if applicable, and caffeine ingestion as normal. For the initial visit, upon arrival to the “laboratory,” participants provided written informed consent as described above. At subsequent visits, participants were reminded of protocol details and asked for verbal confirmation of their continued interest in the study. A schematic of the protocol is shown in Figure 2. Participants rested supine for at least 15-min before vascular measures were conducted. During this period, participants were instrumented with ECG-electrodes, asked questions to confirm standardization adherence, and a toenail sample was collected. Gas exchange measures were assessed after a minimum of 10 min of supine rest followed by a minimum of two blood pressure measurements. Vascular measures, pulse wave velocity (PWV), followed by flow mediated dilation (FMD), were conducted thereafter. Anthropometric variables (height, weight, and waist circumference) were collected while standing. The participant was then seated for collection of a venous blood sample. A questionnaire was administered followed by impulse oscillometry testing. Finally, the participant was accompanied by a researcher to a separate room to complete spirometry tests followed by a sputum induction protocol. The entire testing protocol lasted 90–120 min in total. The same protocol sequence was followed for post-season measures, with time of day matched to the individual's pre-season timepoint whenever feasible. Participants were asked to adhere to the same standardizations as their pre-season testing.

The same measures will be collected each season, with the addition of new methods and analyses based on forthcoming collaborations, BCWS research priorities, and future publications prompting emerging health research aims in WFFs. Some of these future methods and rationale following the first year of the CREWS Study are outlined in detail in the discussion.

### Experimental overview—Acute studies

**Figure 3:**
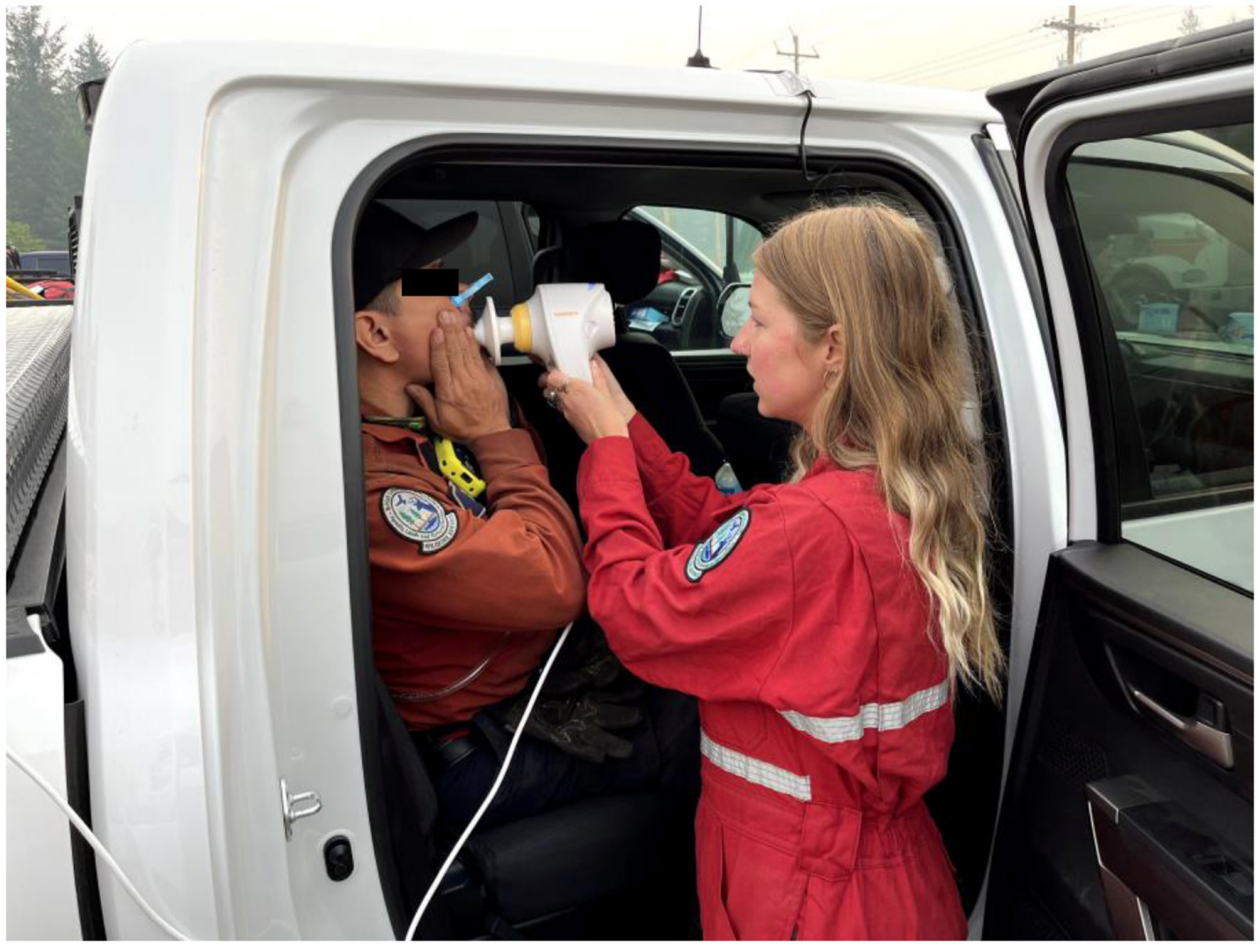
A researcher performs impulse oscillometry at staging in the back of a BCWS vehicle.

Due to time constraints on the WFFs, research team capacity, and uncontrolled variables related to the wildfire environment, it is infeasible to conduct the entire longitudinal protocol in the field. Instead, acute studies focus on cardiorespiratory measures which can be pragmatically applied in the field setting, before and after a single shift, to support existing health research conducted in WFFs. Moreover, acute studies are adapted each season building upon the previous season's findings and emerging research priorities between key partners when applicable. Participation in midseason testing is based on opportunistic sampling of individuals already enrolled in the longitudinal study to avoid performing the detailed informed consent process in the field. To arrange midseason testing, BCWS R&I closely tracks deployment assignments of the crews enrolled in the study and organizes field testing based on research team availability and geolocation of crew deployments. For example, in 2024, we conducted selected midseason respiratory measures (gas exchange and impulse oscillometry) from July to August during 2–4 days across five different deployments (see Figure 1). On testing days, we recruited 2–4 individuals to complete gas exchange and impulse oscillometry measures in the morning at the staging area (see Figure 3), followed by outfitting with air sampling equipment. When safe and feasible to do so, the research team followed participants to the fire line to take detailed field notes about daily tasks and RPE use. At the end of the day, air sampling equipment was collected, and the same respiratory measures were conducted again. In cases where direct observation of the participant was not possible, due to safety reasons or otherwise, we relied on participant recall from that day to recount task engagement and RPE use. To avoid interference with participants' normal workday routine and habits, we did not ask participants to adhere to standardizations for the acute studies.

### Anthropometric variables

Anthropometric variables (height, weight, waist circumference, systolic and diastolic blood pressure) were collected at the pre- and post-season timepoints according to standard techniques (See Supplementary material 1, *Anthropometric Variables*).

### Questionnaire data—Longitudinal study

Questionnaires were conducted in the longitudinal study using survey software (Qualtrics, Provo, UT) to obtain demographic, relevant history, and behavior information. Items included participant date of birth, racial origin/lineage, sex, menstrual cycle phase and hormonal contraceptive use if applicable. Smoking, other tobacco, nicotine, and marijuana use/history were collected. Medication and supplement use was documented. Pertinent medical history including known (diagnosed) cardiovascular, respiratory, cerebrovascular, metabolic, renal, or hepatic diseases were recorded. Firefighting history was also collected detailing number of years worked in any firefighting sector (e.g., structural, wildland, etc.) and average number of months worked per year. Typical use of RPE was documented in addition to respiratory symptoms. Respiratory event risk has been shown to be predicted by symptoms such as dyspnea, chronic mucus secretion, wheezing and cough when clinical indicators of pulmonary function (FEV_1_/FVC) are normal (>0.7), highlighting the value in recording symptoms in addition to clinical measures of lung function (50). In the post-season questionnaire questions regarding exact number of fire line days, number of days since last fire line day, and general RPE use were added. In subsequent seasons, information regarding off-season occupation and general activity to account for ongoing exposure was recorded.

### Questionnaire data—Acute studies

During mid-season data collection, paper questionnaires were given to participants at the staging area to complete. Members of the research team transcribed paper questionnaires into an electronic version (Qualtrics, Provo, UT). On the first encounter (deployment) with a specific crew, two questionnaires were administered to every present crew member enrolled in the longitudinal study. Both questionnaires detailed task-engagement, RPE use, smoke perception, and health symptoms, with one survey specific to the current deployment (Supplementary material 2) and one specific to the entire season (Supplementary material 3). During each subsequent deployment where air sampling was conducted, only the questionnaire specific to the current deployment was administered to every present crew member within the longitudinal study.

### Measures of respiratory function

All respiratory function methods are described in detail in Supplementary material 1.

#### Spirometry

Spirometry was performed according to the 2019 American Thoracic Society and European Respiratory Society's guidelines to obtain clinical indices of pulmonary function (e.g., forced expiratory volume in 1 second, FEV_1_; forced vital capacity, FVC; FEV_1_/FVC, mid-expiratory flow; peak expiratory flow; forced expiratory flow at 50%) (51).

#### Oscillometry

Impulse oscillometry was used to assess total and peripheral airway impedance via the measurement of airway resistance and reactance. Impulse oscillometry measurements were conducted in accordance with current technical standards outlined by the European Respiratory Society (52) concurrent with manufacturer's instructions (Tremoflo C-100 Airwave Oscillometry System, Thorasys, Montreal, Quebec, Canada) prior to spirometry and sputum induction protocols.

Impulse oscillometry is a sensitive and reliable measure of airway function, which is increasingly used in the diagnosis of airway diseases (53, 54). For example, total airway reactance generated from impulse oscillometry can be used in the diagnosis of asthma, even in cases where spirometry is preserved (normal FEV_1_) (53). Impulse oscillometry abnormalities were also found in individuals who smoked <20 pack-years despite preserved spirometry, but in those with increased smoking frequency, impulse oscillometry provided similar insight of respiratory insult as spirometry (55). This suggests that impulse oscillometry may provide early diagnostic insight of the respiratory system prior to traditional clinical markers of pulmonary function.

#### Pulmonary gas exchange

A validated noninvasive pulmonary gas exchange monitor (MediPines Gas Exchange Monitor; MediPines Corp., Yorba Linda, CA) was used to collect heart rate (HR; from pulse oximetry), peripheral oxygen saturation (SpO_2_), alveolar partial pressure of oxygen (P_A_O_2_), and estimated arterial partial pressure of oxygen (P_a_O_2_) (56). An “oxygen deficit” is calculated as the difference between P_A_O_2_ and P_a_O_2_. This assessment of gas exchange—similar to the alveolar to arterial O_2_ gradient—has been validated across healthy and patient populations (57, 58).

### Measures of vascular function

The methods employed to assess vascular function are described in detail in Supplementary material 1.

#### Flow mediated dilation

Reactive hyperemia flow mediated dilation (RH-FMD) of the brachial artery was used to assess endothelium-dependent vasodilatory function according to international guidelines (59). A high-resolution duplex ultrasound (Terason uSmart 3,300, Teratech) with a 10-MHz multifrequency linear array probe (15L4 Smart Mark, Teratech) was used to simultaneously measure diameter and blood velocity (insonation angle, 60°) of the distal third of the left brachial artery.

RH-FMD non-invasively assesses endothelium-dependent vasodilatory function which is blunted with vascular aging and in various patient populations such as coronary artery disease and hypertension (60, 61). FMD is also increasingly used as an indicator of clinical risk (59). For example, in 3,026 healthy adults, cardiovascular event free survival was ~5% higher in those with an FMD greater than the sex-specific medians (4.2% females and 3.6% males) compared with those at or below the median (62). In conjunction with traditional predictive tools of cardiovascular risk such as the Framingham Risk Score, FMD better reclassifies individuals into low-, intermediate-, or high-risk groups than Framingham Risk Score alone (62). This suggests that FMD may be more sensitive to subclinical cardiovascular disease when combined with traditional measurements.

#### Pulse wave velocity

The PWV between the carotid and femoral artery is the gold standard for the non-invasive assessment of central arterial stiffness and was conducted according to current expert consensus (63). A hand-held tonometer (SPT-301; Millar Instruments, Houston, TX, USA) was used to sequentially measure pressure at the carotid and femoral sites concurrent to a lead-III echocardiogram (FE 132; ADInstruments, Colorado Springs, CO, USA).

Carotid-femoral PWV (cf-PWV) provides a measurement of central arterial stiffness naturally associated with aging, but premature rigidity of the vessels is also associated with atherosclerosis (64). cf-PWV is also used as a predictive tool to for large artery (aortic) stiffness and is thereby predictive of cardiovascular-related and all-cause mortality (65). In healthy individuals, PWV increases with age, whereby from 30 to 70 years of age, PWV increases from 6.5 m/s, 7.2 m/s, 8.3 m/s, and 10.3 m/s respectively, with each subsequent decade (64). In the presence of traditional cardiovascular disease risk factors such as grade II/III hypertension, these values are further increased (8.2 m/s, 9.8 m/s, 10.5 m/s, and 12.2 m/s), suggesting their diagnostic value (64). In a pool of 1,325 individuals, cf-PWV of >12 m/s had a higher incidence rate of major adverse cardiovascular events and all-cause mortality (66). Furthermore, Cox proportional hazard ratios of 1.31 indicate that in individuals 65 years old or younger, cf-PWV was an independent risk factor for major adverse cardiovascular events (66). Therefore, cf-PWV in the current study provides a subclinical value related to vascular function.

### Circulating and airway-specific inflammation

Venous blood was collected and processed for future exploratory analysis of established circulating markers involved in vascular (67–69) and pulmonary (70–74) inflammatory pathways and injury. Specific details of biomarkers are presented in Table 2.

**Table 2:** Circulating and airway-specific biomarkers.

**Biomarker**	**Study component**	**Specific function**	**References**
**Blood biomarkers**			
Interleukin-6 (IL-6)	Cardiovascular	Systemic inflammation	(68, 73)
Interleukin-8 (IL-8)	Cardiovascular	Systemic inflammation	
Tumor necrosis factor alpha (TNF-α)	Cardiovascular	Systemic inflammation	
Intercellular adhesion molecule-1 (ICAM-1)	Cardiovascular	Vascular inflammation	
Vascular cell adhesion molecule-1 (VCAM-1)	Cardiovascular	Vascular inflammation	
C-reactive protein (CRP)	Cardiovascular	Systemic inflammation	(67)
Vascular endothelial-cadherin (VE-Cadherin)	Cardiovascular	Vascular inflammation	(155)
Vascular endothelial growth factor receptor 2 (VEGFR2)	Cardiovascular	Vascular inflammation	(69)
Surfactant protein D (SP-D)	Respiratory	Pulmonary inflammation	(72)
Matrix metalloproteinase-9 (MMP9)	Respiratory	Pulmonary inflammation	(71)
Club cell secretory protein 16 (CC16)	Respiratory	Pulmonary inflammation and oxidative stress	(70)
Monocyte chemoattractant protein-1 (MCP-1)	Respiratory	Systemic inflammation	(74)
**Sputum biomarkers**			
Interleukin-4 (IL-4)	Respiratory	Mast cell inflammation	(76)
Major basic protein (MBP)	Respiratory	Eosinophilic inflammation	(75)
Eosinophil cationic protein (ECP)	Respiratory	Eosinophilic inflammation	(78)
Myeloperoxidase (MPO)	Respiratory	Neutrophilic inflammation	(79)
Eotaxin-1 (CCL11)	Respiratory	Allergic response cell inflammation	(77)

Sputum samples were collected and supernatant isolated for the analysis of relevant markers of inflammatory cell differential (see Table 2) (75–79). A 230 V~50 Hz ultrasonic nebulizer (Universal III, Flaem Medical Devices, Italy) was used to vaporize hypertonic (7%) saline for inhalation to induce sputum production which was collected and stored in −20°C for short term (< 4-weeks) storage. Sputum plugs were then separated, weighed, washed and treated, with the remaining supernatant saved and stored at −80°C for future analysis of biomarkers. Multiplex (e.g., Bio-Plex Multiplexing, Mesoscale Discovery) and enzyme-linked immunoassays will be used to assess samples for serum and sputum supernatant markers described above.

### Exposure assessments

#### Toenail samples

Human toenail samples were collected pre- and post-season for the analysis of toxic metal exposure by inductively coupled plasma mass spectrometry (ICP-MS) (80). Toenails, due to their slow growth rate, provide stable and reliable information on exposures of the preceding ~7–12 months. Human toenails have previously been used to demonstrate high exogenous arsenic exposure in individuals living near former mine sites and high levels of cadmium in welders compared with control groups (81, 82). Although there is evidence of dangerous heavy metals in flame retardant material which is commonly in contact with WFFs (83), this exposure has not been previously addressed from toenail samples.

#### Air monitoring

For each midseason sampling day, 2–4 WFFs per day were selected for air monitoring, which involved wearing a personal sampling pump (GilAir Plus, Sensidyne Ltd.) and carbon monoxide detector (Tango TX-1, Industrial Scientific). The personal air pump collected respirable particulate (with an aerodynamic diameter cut-point of 4 μm) via NIOSH 0600 and respirable crystalline silica, as alpha quartz, via NIOSH 7,500, while carbon monoxide was assessed according to NIOSH 6,604 (84).

All filters were analyzed by an American Industrial Hygienists Association-accredited industrial hygiene laboratory (EMSL Analytical Inc., Mississauga, ON). The research team accompanied WFF crews from the staging area to the fire line to ensure that air monitoring equipment was functioning properly and to collect field observations regarding environmental characteristics, fire behavior, RPE use, and task engagement. The research team attempted to follow crew members wearing monitoring equipment throughout their shift. Individual WFFs were sometimes assigned to different areas of the fire. As a result, the research team was not always able to keep continuous records on everyone, and instead relied on regular (every 1–3 h) radio check-ins and subject recall for daily task engagement and RPE use.

### Statistical plan

Statistical analyses will be performed using RStudio software (The R Foundation for Statistical Computing, R version 4.3.3, 2024) and GraphPad Prism software (Version 10.3.1, 2024). The distribution of the data will be assessed via Shapiro-Wilk test and by visual inspection of Q-Q plots. Homogeneity of variance will be determined by Levene's test. For the longitudinal study, linear (parametric) and generalized (non-parametric) mixed models for repeated measures will be used to assess various outcomes as fixed effects across timepoints to account for non-independent observations. To account for individual variation in the repeated measurees design, participant identification will be included as a random effect. Covariates for firefighting experience in the longitudinal study and exposure variables (PM_4_, silica, and carbon monoxide) and RPE use in the acute studies will also be applied to these models to account for variability in current and historical exposure. Paired *t*-tests will be used to determine cross-shift changes in various outcomes. Pearson's (parametric) or spearman's (non-parametric) correlations will be used to determine relationships between exposure and cardiorespiratory outcomes.

## Anticipated results

A comprehensive list of anticipated results from the CREWS Study are provided in Table 3. Projected outcomes for forthcoming data collection periods (2025 and 2026) are highlighted in italics.

**Table 3:** Anticipated results from the CREWS study.

**Measurement name**	**Study component**	**Outcome metric**	**Acute and/or longitudinal**	**Study interval**						
				**2024**			**2025**		**2026**	
				**Pre**	**Mid**	**Post**	**Pre**	**Post**	**Pre**	**Post**
**Anthropometric data**										
Height	General	Clinical	L	Y	N	Y	*Y*	*Y*	*Y*	*Y*
Weight	General	Clinical	L	Y	N	Y	*Y*	*Y*	*Y*	*Y*
Body mass index	General	Clinical	L	Y	N	Y	*Y*	*Y*	*Y*	*Y*
Waist circumference	General	Clinical	L	Y	N	Y	*Y*	*Y*	*Y*	*Y*
Systolic blood pressure	General	Clinical	L	Y	N	Y	*Y*	*Y*	*Y*	*Y*
Diastolic blood pressure	General	Clinical	L	Y	N	Y	*Y*	*Y*	*Y*	*Y*
**Questionnaire data**										
Age	General	Covariate	Both	Y	N	Y	*Y*	*Y*	*Y*	*Y*
Medical history	General	Covariate	L	Y	N	Y	*Y*	*Y*	*Y*	*Y*
Firefighting history	Exposure	Covariate	L	Y	N	Y	*Y*	*Y*	*Y*	*Y*
Occupation history	Exposure	Covariate	L	Y	N	N	*Y*	*Y*	*Y*	*Y*
Respiratory protective equipment behaviors	Exposure	Covariate	Both	Y	Y	Y	*Y*	*Y*	*Y*	*Y*
Health symptoms	General, Respiratory, Cardiovascular	Clinical	Both	Y	Y	Y	*Y*	*Y*	*Y*	*Y*
Smoke perception	Exposure	Qualitative	A	N	Y	N	*N*	*N*	*N*	*N*
Task engagement	Exposure	Qualitative	A	N	Y	N	*N*	*N*	*N*	*N*
**Spirometry**										
Forced expiratory flow in 1 s (FEV_1_)	Respiratory	Clinical	L	Y	N	Y	*Y*	*Y*	*Y*	*Y*
Forced vital capacity (FVC)	Respiratory	Clinical	L	Y	N	Y	*Y*	*Y*	*Y*	*Y*
FEV_1_/FVC	Respiratory	Clinical	L	Y	N	Y	*Y*	*Y*	*Y*	*Y*
Mid-expiratory flow (MEF)	Respiratory	Clinical	L	Y	N	Y	*Y*	*Y*	*Y*	*Y*
Peak-expiratory flow (PEF)	Respiratory	Clinical	L	Y	N	Y	*Y*	*Y*	*Y*	*Y*
Forced expiratory flow at 50% (FEF_50_)	Respiratory	Clinical	L	Y	N	Y	*Y*	*Y*	*Y*	*Y*
**Impulse oscillometry**										
Total airway resistance (R_5_)	Respiratory	Subclinical	Both	Y	Y	Y	*Y*	*Y*	*Y*	*Y*
Peripheral airway resistance (R_5 − 20_)	Respiratory	Subclinical	Both	Y	Y	Y	*Y*	*Y*	*Y*	*Y*
Total airway reactance (AX)	Respiratory	Subclinical	Both	Y	Y	Y	*Y*	*Y*	*Y*	*Y*
Peripheral airway reactance (X_5_)	Respiratory	Subclinical	Both	Y	Y	Y	*Y*	*Y*	*Y*	*Y*
**Pulmonary gas exchange**										
Oxygen deficit (P_A_O_2_-P_a_O_2_)	Respiratory	Subclinical	Both	Y	Y	Y	*Y*	*Y*	*Y*	*Y*
Estimated partial pressure of arterial oxygen (P_a_O_2_)	Respiratory	Subclinical	Both	Y	Y	Y	*Y*	*Y*	*Y*	*Y*
Partial pressure of alveolar oxygen (P_A_O_2_)	Respiratory	Subclinical	Both	Y	Y	Y	*Y*	*Y*	*Y*	*Y*
End-tidal carbon dioxide (P_ET_CO_2_)	Respiratory	Subclinical	Both	Y	Y	Y	*Y*	*Y*	*Y*	*Y*
Oxygen Saturation (SpO_2_)	Respiratory	Clinical	Both	Y	Y	Y	*Y*	*Y*	*Y*	*Y*
**Flow mediated dilation**										
Reactive hyperemia flow mediated dilation (RH-FMD)	Cardiovascular	Subclinical	L	Y	N	Y	*Y*	*Y*	*Y*	*Y*
**Pulse wave velocity**										
Resting heart rate (bpm)	Cardiovascular	Subclinical	L	Y	N	Y	*Y*	*Y*	*Y*	*Y*
Central pulse transit time (s)	Cardiovascular	Subclinical	L	Y	N	Y	*Y*	*Y*	*Y*	*Y*
Carotid-femoral pulse wave velocity (cf-PWV)	Cardiovascular	Subclinical	L	Y	N	Y	*Y*	*Y*	*Y*	*Y*
**Inflammatory biomarkers**										
Blood biomarkers	Cardiovascular and Respiratory	Mechanistic	L	Y	N	Y	*Y*	*Y*	*Y*	*Y*
Sputum biomarkers	Respiratory	Mechanistic	L	Y	N	Y	*Y*	*Y*	*Y*	*Y*
**Exposure assessments**										
Heavy metal profile (Cadmium, chromium, vanadium, manganese, aluminum, mercury, lead) from toenail clippings	Exposure	Biological exposure	L	Y	N	Y	*Y*	*Y*	*Y*	*Y*
Respirable crystalline silica mass concentration	Exposure	Air sample	A	N	Y	N	*N*	*N*	*N*	*N*
Respirable particulate matter (PM_4_) mass concentration	Exposure	Air sample	A	N	Y	N	*N*	*N*	*N*	*N*
Carbon Monoxide (CO)	Exposure	Air sample	A	N	Y	N	*N*	*N*	*N*	*N*
**Forthcoming assessments**										
Fractional exhaled nitric oxide (FENO)	Respiratory	Mechanistic	Both	N	N	N	*Y*	*Y*	*Y*	*Y*
Heart Rate Variability	Cardiovascular	Subclinical	L	N	N	N	*Y*	*Y*	*Y*	*Y*
Gut microbiome profile	Gastrointestinal	Subclinical	L	N	N	N	*Y*	*Y*	*Y*	*Y*
Carboxyhemoglobin saturation (SpCO)	Exposure	Biological exposure	Both	N	N	N	*Y*	*Y*	*Y*	*Y*
In-field physiological variables: heart rate, ventilation rate, actigraphy, core temperature	Exposure	Biological exposure	A	N	N	N	*N*	*N*	*N*	*N*
Central nervous system biomarkers	Brain	Mechanistic	L	N	N	N	*N*	*N*	*Y*	*Y*

L, longitudinal study; A, acute study; Pre, pre-season; Mid, mid-season; Post, post-season; Y, yes; N, no. Projected results for future data collection periods presented in italics.

## Discussion

Although the CREWS Study will be the first to comprehensively address the cardiorespiratory health effects of wildland firefighting across three consecutive seasons, the WFFEHE was a seminal study which previously attempted some shared aims in 2018 (47). Unfortunately, due to the coronavirus disease 2019 pandemic, the WFFEHE study ended in 2019, prior to its 3-year goal. Nevertheless, during the 2018 and 2019 seasons, more than 150 WFFs, most of whom had previous wildland firefighting experience, from six U.S. Interagency Hotshot crews across Colorado and Idaho were tested for various cardiorespiratory measures including central blood pressure and PWV estimates, serum markers of inflammation, spirometry, and fractional exhaled nitric oxide. Fifty-six of these individuals were tested pre- and post-season for both seasons (4 timepoints) and the majority of these participants (75 WFFs) participated in a single season of testing. The CREWS Study assesses several biomarkers and indices identical to that of the WFFEHE study. For example, spirometric indices (FEV_1_, FVC, peak expiratory flowrate, and the FEV_1_/FVC), as well as serum biomarkers of inflammation (surfactant protein-D, tumor necrosis factor alpha, IL-8, IL-6, matrix metalloproteinase 9, club cell protein-16, and monocyte chemoattractant protein 1) are investigated across both studies. PWV was also reported in the WFFEHE study, albeit using sub-standard techniques, which are discussed in detail below. Crossover of exact metrics between the two studies will add unique power across heterogenous cohorts.

To date, only two studies from the WFFEHE study have been published. Navarro et al. reported exposures to volatile organic compounds across 3-days of midseason wildfire suppression, where benzene, toluene, ethylbenzene, xylene were higher on days where chainsaw work was common and formaldehyde and acetaldehyde were highest on a firing operation day where ambient smoke levels were generally high (47). These compounds are known to induce respiratory irritation and neurological impairment and some are carcinogenic (e.g., benzene, formaldehyde, and acetaldehyde) (85, 86), although health outcomes were not reported with this study. Urinary levoglucosan, a metabolic marker of biomass combustion exposure, was also observed to be highest on a day where WFFs were exposed to smoldering vegetation (e.g., mop-up), and was increased in 65% of all paired samples across the 3 days (47). Together, these findings indicate the complex exposures associated with wildland firefighting, particularly across different tasks, and solidify a need to identify concurrent health consequences. In the another WFFEHE study, adverse health behavior changes were reported during two fire seasons such as increased smokeless tobacco use, binge drinking (alcohol) on days off, and sugar-sweetened beverage consumption in crews (87). Heterogeneity of the substance used varied at the crew-level, indicating diverse sociocultural paradigms amongst individual crews that should be taken into consideration when developing mitigation strategies (87). To our knowledge, the cardiorespiratory health data collected during the WFFEHE study has not been published at the time of preparation of this manuscript.

The WFFEHE study is complementary to the study herein but includes a less detailed approach to the potential cardiorespiratory consequences associated with wildfire suppression. However, key differences in physiological measures, addressing an ongoing need for long-term cardiorespiratory research in a diverse Canadian WFF workforce, necessitates the extended aims of the CREWS Study. The following discusses the evidence and rationale for the ongoing comprehensive assessment of WFF health employed by the CREWS Study.

### Respiratory health in WFFs

Spirometry is perhaps one of the most widely applied measurements of pulmonary function conducted in WFFs (32, 33, 35, 38, 49, 88–91). The FEV_1_ has been shown to be reduced by approximately 0.22 L/s cross-shift (34, 38, 49, 88, 90) and 0.217 L/s cross-season (32–34, 49) in WFFs. Although normal diurnal variation has been reported across a shift (89), each additional day of prescribed burn exposure was associated with further declines in the pre-shift FVC and FEV_1_, suggesting a cumulative exposure effect on lung function. We hypothesize that a similar finding will emerge in the CREWS Study whereby declines in spirometric lung function will be observed post-season with cumulative reductions across each season. Furthermore, the current participant pool includes a broad and realistic range of wildland firefighting experience and will continue to expand through the open enrollment design. Thus, the influence of short and long-term wildland firefighting on lung function may be determined.

We complemented spirometry measurements, also applied in the WFFEHE study, with impulse oscillometry, a subclinical measure that provides regional specificity and precedes clinical manifestation with its high sensitivity to lung mechanics (92, 93). Two recent studies have employed impulse oscillometry in the context of wildfire smoke exposure. One found that area under the reactance curve was increased during a pollution period (e.g., fire season) in 16 Thai WFFs compared with 12 controls, suggesting a short-term effect of occupational pollution exposure on the elastic properties of the lung (91). In another study, airway reactance at the end of inspiration and expiration was acutely reduced in children but not adults several days after a nearby controlled burn period (94), implying dysfunction of the elastic properties of the lung. Impulse oscillometry has not been examined acutely in WFFs but may provide additional subclinical context of peripheral airway mechanics related to spirometric declines in lung function commonly described throughout the literature.

Smoke, dust, and ash particulates can lodge deep into the gas-exchanging regions of the airways and the capillaries potentially effecting diffusion factors related to surface area of the membrane, alveolar pressure difference, or membrane thickness through direct deposition or edema (95). In 169 non-smoker firefighters without chronic respiratory disease involved in several weeks of wildfire suppression during a single large wildfire event (2016 Fort McMurrary, Alberta), enhanced bronchial wall thickening assessed via high resolution chest computerized tomography was observed in 21.3% of individuals (36). Nodules were also noted in 31.3% of this cohort, suggesting a fibrotic effect of wildfire exposure. In a similar cohort, 100 of these individuals self-reported ongoing symptoms related to their involvement with the fire event, demonstrating a reduction in observed to predicted ratio for diffusion capacity of the lung in individuals with the highest estimated PM_2.5_ exposure. This observation indicates a relationship between smoke exposure and gas exchange impairment (36). This study, however, relied on exposure estimates of a combination of vegetative and synthetic fuel sources and may not represent cumulative seasonal exposures by WFFs given both structural and WFFs were included. As planned in the current CREWS Study, further exploration of the type of smoke exposure using gold standard air monitoring methods and its impact on gas exchange in response to wildfire suppression efforts are needed to fully characterize these effects.

### Vascular health in WFFs

The subclinical vascular function measures applied in the CREWS Study require experienced technicians, adequate rest, and controlled environments to accurately conduct. In a 2-h controlled human exposure study to concentrated ambient fine particles (PM_2.5_, 150 μg/m^3^) and ozone (120 ppb), no difference in FMD was measured despite reductions in brachial artery diameter elicited by the pollution condition in healthy adults (96). Similarly, in structural firefighters exposed to a high concentration of woodsmoke (1 mg/m^3^) for 1-h with intermittent exercise, vascular vasomotor function of fibrinolytic function was not affected as assessed via FMD and vasoactive substance infusion (40). In another cross-sectional study, FMD was measured in individuals exposed for 6-weeks to a large coal fire 4-years after the incident; FMD was unchanged compared with the unexposed group (97). The findings from these studies may not necessarily reflect a lack of influence of pollutants on the vasculature; rather, these are reflective of the composition, concentration, and acute nature of a single, controlled exposure or narrow window of exposure. Thus, a need remains to directly assess chronic changes in endothelial function in WFFs.

As employed in this study, the gold standard non-invasive method for PWV is tonometry; however, algorithm-based systems such as the Mobil-O-Graph used in the WFFEHE study are less reflective of central arterial stiffness and should be cautioned as mere estimates with limited accuracy (63, 98). PWV has been demonstrated to increase by ~50% after a controlled 3-h exposure to 314 μg/m^3^ of woodsmoke with intermittent exercise compared with filtered air (39). PWV is also increased by 0.1–0.2 m/s 24-h following 2-h of exposure to various PM_2.5_-emitting cookstoves, ranging from 10 to 500 μg/m^3^, compared with a control condition, suggesting an association between PM_2.5_ and arterial stiffness (99). Arterial stiffness is exacerbated by the presence of traditional cardiovascular risk factors like hypertension (64). For example in young adults (< 30 years), grade II/III hypertension was associated with an increased PWV (+1.6 m/s) from the optimal value (6.1 m/s), which increases further with advancing age (64). Given recent evidence suggesting significantly higher odds (odd ratio: 2.84) of developing hypertension as a WFF compared with the general population, PWV may highlight a mediator of the observed increase in blood pressure in WFFs. Arterial stiffness, given its power to predict cardiovascular risk, may be a harbinger to the development of hypertension (64, 100), necessitating detailed investigation in WFFs with gold standard methodology.

### Mechanisms of wildfire-related cardiorespiratory dysfunction

An emerging mechanistic link between wildfire-associated pollution and cardiorespiratory decline is inflammation, which can also present with oxidative stress (101, 102). Namely, proinflammatory pathways may be activated by particulates and other toxic components of wildfire smoke, dust, and ash, resulting in dysfunction of the airways (37, 46, 91, 103–105). Moreover, this inflammation can permeate systemically, into the circulation, thereby influencing a “spillover” effect within the cardiovascular system (104, 105). Circulating inflammatory cytokines such as IL-8, IL-6 and IL-10 are modulated across a single, ~12-h wildfire suppression shift (103, 105) in addition to vascular-specific inflammatory markers like C-reactive protein and intercellular adhesion molecule-1 (103). The characterization of airway specific inflammation is also exhibited through neutrophilic and eosinophilic activity in the bronchoalveolar lavage fluid of firefighters who participated in wildfire suppression for at least several continuous days in a season (104). Oxidative pathways, which are intertwined with—and activating of inflammatory pathways (101)—have also been demonstrated to be affected by wildfire smoke exposure. This is evidenced by cross-shift changes in the oxidative stress marker, malondialdehyde paired with polycyclic aromatic hydrocarbon exposure marker, 1-hydroxypyrene (20, 106). Cross-sectional evidence also exhibits that a higher augmentation index (arterial stiffness) is associated with increased oxidative stress in WFFs, suggesting potential biomarkers of vascular function in this cohort (46). In the current study, the dichotomization of inflammation specific to the circulation (blood) vs. the airways (sputum) across multiple seasons concurrent with cardiorespiratory outcomes, may further elucidate the nature and temporal pattern of cardiorespiratory disease progression in wildfire personnel.

### Exposures of wildfire suppression

PM_2.5_, as a primary constituent of wildfire smoke, is commonly reported with wildfire exposure research. The National Institute for Occupational Safety and Health (NIOSH) has recently identified PM_2.5_ as the single major hazard of concern present in wildfire smoke (107). Recent evidence from 1999 to 2012 in California suggests that wildfire-specific PM_2.5_ results in increased respiratory hospital emissions compared with PM_2.5_ from other sources [e.g., vehicular emissions, sulfate and nitrate aerosols, soil, agricultural, and industrial emissions (108)]. PM_4_, although slightly larger than PM_2.5_, can also penetrate deep into the airways and indirectly effect the cardiovascular system (109). Although PM_4_ encompasses wildfire smoke (PM_2.5_), it also includes larger dust/soil and ash particles, which are more representative of the complex occupational exposures of wildfire suppression (10). Mean PM_4_ concentrations of 2.56 mg/m^3^, 1.22 mg/m^3^, and 1.08 mg/m^3^ have been observed for specific fire line tasks involving direct suppression, mop-up, and holding respectively, which exceed a proposed WFF-specific interim occupational exposure limit of 0.7 mg/m^3^ (10, 11, 110, 111). It is worth noting, that these concentrations also tend to be much higher than controlled human exposure studies of PM_2.5_, where participants are exposed for much shorter periods of time (1–3 h) compared with occupationally (>12 h). Respiratory crystalline silica, evident in PM_4_, represents a soil-derived mineral exacerbated during soil-disturbing tasks commonly conducted by WFFs (110). It is a common occupational hazard in mining, construction, agriculture, and the engineered stone industries (112). Chronic exposure to silica can cause lung cancer and silicosis, a permanent, and irreversible fibrotic lung disease (113). As a result, airborne concentrations of silica in workplaces are strictly controlled to very low limits (ACGIH: < 0.025 mg/m^3^) (112). Respirators, engineering controls such as local exhaust ventilation and dust control methods, and the medical surveillance of exposed workers can be used in workplaces to protect worker health, but are not always feasible in the wildland firefighting occupation (114). Carbon monoxide (CO) is also released by biomass combustion and is a prominent byproduct of two-stroke engines, such as chainsaws and pumps commonly used for wildland firefighting operations (115). Together, PM_4_, respiratory crystalline silica, and CO, more comprehensively address the complex and task-specific exposures experienced on the fire line which are not limited to smoke.

Human toenail samples were collected to assess toxic metal exposure associated with wildfire suppression by ICP-MS, a technique previously used to assess hazardous exposures in other occupational cohorts (81). Carcinogenic metals such as chromium, which can be derived from soil and ash, have been shown to be exacerbated during wildfire season (116). Similarly, other hazardous metals such as aluminum, iron, potassium, titanium, and manganese were consistently elevated on smoke-impacted days in California from 2006 to 2018 (117). Extremely high levels of chromium, cadmium, and vanadium in fire retardant were recently reported, suggesting another source of exposure common to the work zones of WFFs (83). Although urinary analysis has revealed toxic metal exposures in WFFs, it is only representative of acute exposures to such hazards (80). The characterization of these metals in toenails, many of which are recognized as neurotoxicants (manganese, lead, aluminum), carcinogens (chromium, cadmium) or contributors to cardiovascular disease (cadmium, lead, mercury), may help characterize health risk in WFFs and ultimately inform mitigation strategies.

### Study strengths

#### Integrated knowledge translation

**Figure 4:**
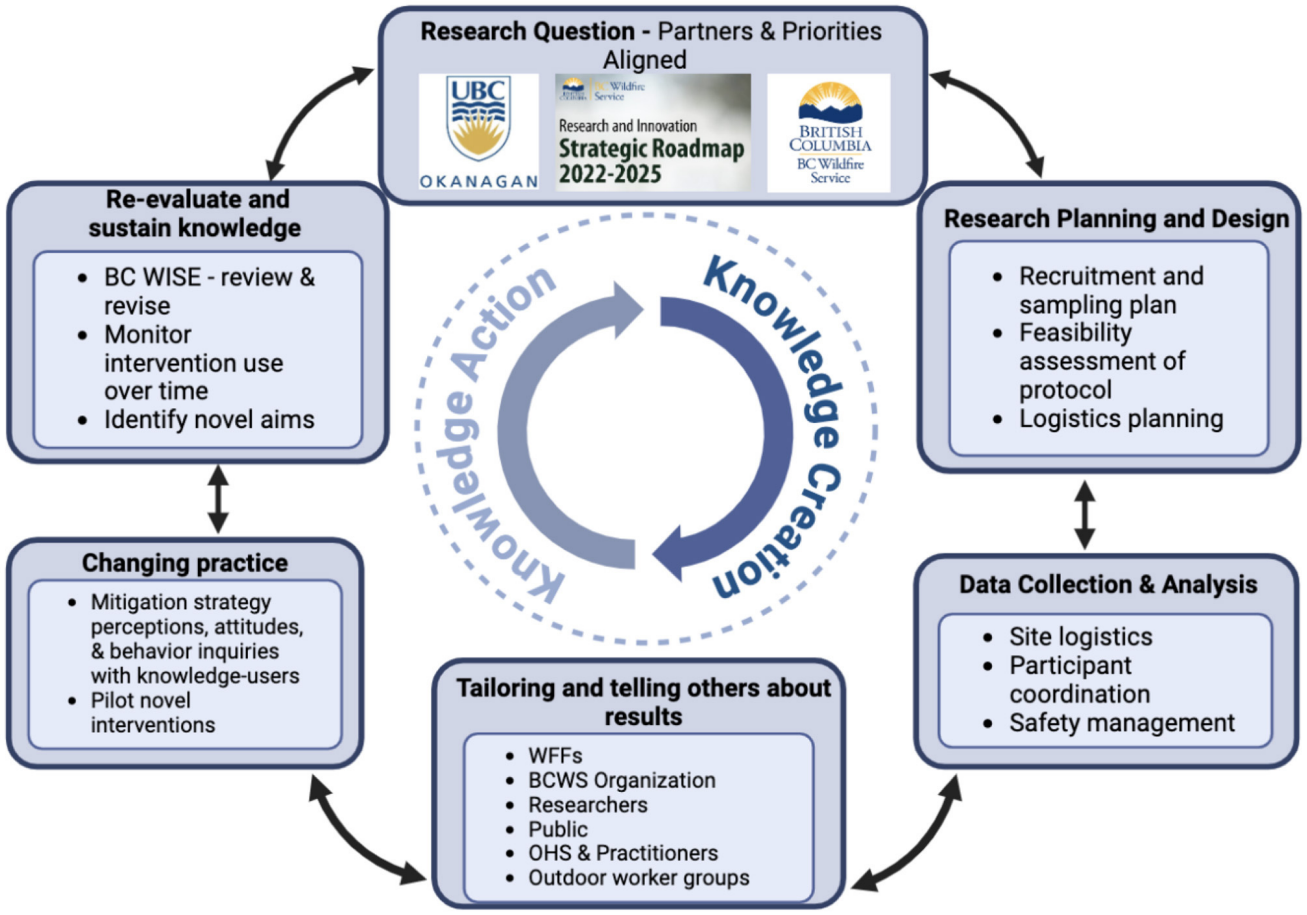
Flow chart exemplifying the integrated knowledge translation of the CREWS study. Knowledge-users (BCWS) are equal partners alongside researchers (UBCO). Research questions are co-developed based on identified gaps and research priorities of both groups. BCWS provides key aspects of research planning and design and facilitates successful data collection and analysis to make knowledge creation possible. Emerging data is tailored to and shared with various knowledge-users (WFFs, BCWS, researchers, public, other outdoor worker groups, and occupational health and safety and practitioners) through annual participant results (WFFs), emailed slide decks and presentations at organization priorities forums (BCWS), peer-reviewed research articles made open-access (public), media releases (public), presentations at FEMA Wildland Quarterly Meetings (OHS and practitioners), and presentations at outdoor worker conferences (Western Forestry Contractors Association conference 2025). Field observations and surveys collect information on behaviors (use), perceptions, and attitudes toward current mitigation strategies (e.g., RPE use) to identify barriers and facilitators of knowledge use. Surveys are also used to collect sentiments about novel interventions and may be piloted within a subset of participants. Ongoing mitigation strategies like RPE use are examined over time to understand compliance and efficacy of the strategy. A steering committee (BC WISE) comprised of knowledge users meets semi-annually to review data and revise research priorities, identifying novel aims, creating guidelines/recommendations, and suggesting new avenues for knowledge translation (dissemination of findings). WFFs, wildland firefighters; BCWS, British Columbia Wildfire Service; OHS, occupational health and safety; BC WISE, British Columbian Wildfire Intervention Steering Entity; RPE, respiratory protective equipment.

With variations in seasonal fire activity and myriad uncontrolled factors, wildland firefighting is an extremely dynamic work environment. This fluidity is further exemplified in the fluctuation of its seasonal workforce, where crew members may change locations, positions, or leave the workforce altogether. Consequently, controlled longitudinal studies are difficult to conduct and rarely described in the literature. To address many of these challenges, we rely on an essential partnership with the BCWS, which began over a year before the initiation of the study. Indeed, a primary strength of the CREWS Study is its integrated knowledge translation (118, 119), involving early and continued co-production of knowledge creation and action with primary knowledge-users (see Figure 4). BCWS R&I—serving as liaisons between UBCO researchers, Canada Wildfire, and the BCWS organization—was closely involved in co-developing research aims to meet BCWS priorities, research planning and design (e.g., recruitment strategy, feasibility consultation, logistical planning), and data collection and analysis (e.g., site logistics, participant coordination, safety management). Although this research is ongoing, BCWS also plays an integral role in knowledge action by helping adapt knowledge to local context so that knowledge can be tailored and shared with diverse knowledge-user groups. Examples of the outputs from co-production of knowledge actions currently include: presenting at internal BCWS priorities forums and external practitioner and occupational health and safety groups (e.g., Federal Emergency Management Agency Wildland Quarterly meetings), individual participant results packets, and local (120, 121) and national (122) media releases. Additionally, informational slide decks have been created for quick internal dissemination of emerging results to the BCWS organization and data has been presented to other outdoor worker cohorts (e.g., Western Forestry Contractors Association Conference, 2025). Forthcoming knowledge translation include peer-reviewed research articles with open-access for public interest. To maintain WFF health, a shared goal between partners, surveys capture perceptions, attitudes, and behaviors of current intervention practices (e.g., RPE use) to better understand barriers and facilitators of knowledge (intervention) use. With forthcoming evidence, novel intervention strategies can be selected, tailored and implemented. Over time, these mitigation strategies can be monitored for uptake and efficacy. Moreover, the implementation of a steering committee of knowledge-users (see below), knowledge can be evaluated, guidelines/recommendations developed, and novel research aims formulated, thereby continuing the iterative cycle of integrated knowledge translation.

#### Future methods

Field-research also provides a unique space in which to observe nuances that may not otherwise be identified without witnessing participants in their occupational environment. Understanding the sociocultural nuances within WFF crews may be essential to effectively implementing future intervention strategies voluntarily adopted by the workforce. The longitudinal, repeated measures design of the CREWS Study also provides an adequate timeframe to develop mutual trust between researchers and participants. Although the longitudinal design of the study will remain constant, the flexibility of the acute midseason studies allows emerging research questions to be addressed to complement and extend our understanding of the health effects of this occupation while meeting the needs of WFFs. Together, these key factors enable the unique perspectives, concerns, and ideas of WFFs *per se* and their occupational environment to be considered, further driving integrated knowledge translation.

Building on this end-user feedback and observations from the first season of experimental testing, updates to data collection on subsequent seasons and participants will include:

##### Fractional exhaled nitric oxide

A measurement used in the WFFEHE study which can be practically applied in the field to quickly and non-invasively assess eosinophilic inflammation of the airways. This measure will be applied in the CREWS Study to complement sputum and serum markers analyzed in the longitudinal study and identify the mechanisms of acute airway dysfunction such as changes in gas exchange.

#### Heart rate variability

Heart rate variability (HRV) is a metric considered to reflect the autonomic control of the heart by assessing the variability of time between consecutive heart beats (123). Moreover, HRV is closely linked with cardiovascular morbidity and mortality with several controlled pollution chamber experiments demonstrating reductions in HRV following acute particulate matter exposure (39, 124). Five minutes of resting electrocardiographic data provide sufficient information to calculate common indices of HRV such as the root mean square and standard deviation of successive normal QRS complexes (NN intervals) (123, 125). Thus, HRV, derived from resting electrocardiogram data obtained during PWV measurements, will provide an added metric to better understand cardiovascular implications of wildfire suppression.

#### Gut microbiome

Evidence suggests that environmental stressors, particularly heat and exercise, can influence the gut microbiome which plays a role in the systemic inflammatory response (126). The co-exposure of heat with smoke, dust, and ash exposure may exacerbate the effects on the gut microbiome, adding to the systemic inflammation and diminishing cardiorespiratory integrity (126). Procedures to assess the gut microbiome in WFFs are forthcoming in our work.

#### Wearable monitors

Wearable devices will be used in the field to collect respiratory rates (127) with air monitoring to determine effective dosages of measured exposures during specific WFF tasks or across the entire shift.

#### Carboxyhemoglobin

A direct measure of carbon monoxide exposure is carboxyhemoglobin, which can be measured in the blood concurrent to air monitoring devices to determine the relationship between breathing zone and physiological concentrations of carbon monoxide. This may be significant in predicting future adverse cardiac events (128) and directly influences vascular function.

#### Discovery biomarkers

The exploratory analysis of exhaled breath condensate cross-season and targeted analysis of cross-shift concentrate based on previously identified markers (non-exchangeable hydrogen types) (90) may be used as biomarkers of exposure and align with changes in cardiorespiratory function. The effect of heat and pollution, given their independent effects on brain health (neuroinflammation and cerebrovascular effects, oxidative stress, and cognitive impairment), is an emerging concern especially in vulnerable populations (129). Discovery panels identifying biomarkers of central nervous system disruption will be applied (subject to funding constraints) to provide exploratory insight into the acute and chronic brain health in WFFs (130).

#### Co-development of interventions

Partners from our diverse team of researchers, clinicians, occupational hygienists, BCWS Research and Innovation Business Area, crew supervisors, and WFFs, will collaborate to form a steering committee (BC Wildfire Intervention Steering Entity; BC WISE) to co-develop a framework with which to annually review data, revise research aims/priorities, identify feasible mitigation strategies, and discuss methods of knowledge translation. Surveys designed to engage primary knowledge-users (WFFs and BCWS leadership) will be used for perceptions and feasibility feedback of proposed intervention methods. Evaluative thinking, or real-time integration of research-based evidence from aims 1–3, will inform the BC WISE framework. The BC WISE will meet semi-annually to discuss recent data from the concurrent study to construct a framework for future aims in terms of improvements, strategy, cohort, budget, and potential partnerships. The BC WISE is currently being formed.

#### Comprehensive approach

The present research initiative also provides a comprehensive assessment of cardiorespiratory health to better understand disease progression in WFFs from the cellular to organismal level. Although the health implications of the wildfire environment are multifaceted and extend beyond that of the cardiorespiratory realm, considering the airway-centric route of exposure, the primary concern indeed relates to the lungs and vasculature. Therefore, in depth assessment are necessary to fully comprehend the etiology of disease and dysfunction. For example, the examination of inflammatory biomarkers in the sputum concurrent to gas exchange assessment, impulse oscillometry, and spirometry, allows a translational approach which consider the mechanisms, subclinical, and clinical manifestation of respiratory dysfunction in WFFs over time, a key strength of the CREWS Study. Applying expansive techniques to cardiorespiratory health allows the identification of biomarkers or subclinical information which can be used for the early detection of disease.

#### Canadian workforce

Many studies regarding WFF health refer to WFFs in the U.S. (10, 32–35, 38, 42, 46, 47, 88–90, 103, 106, 110) or other countries [outside of N America; (20, 49, 91, 104, 105)]. These studies provide an excellent basis of understanding wildfire suppression-associated health effects, but it should be noted that firefighting tactics, biomass fuel sources, and sociocultural behaviors vary regionally, thereby influencing potential health risk. For example, differences in water availability, would cause increased reliance on hand tool use (e.g., grubbing, dry mopping, digging guard/hand-lines), which would, in turn, produce more soil-derived exposures than hosing tactics (e.g., wet lining) (10, 131). Disparate tactics also induce different work rates and thus heat production and ventilation rates, leading to differences in personal exposure concentrations and co-exposure to heat (132). Moreover, regional differences in fuel sources (e.g. grasses vs. forests) may influence divergent exposure hazards amongst WFFs (133, 134). A growing body of health research has increasingly included Canadian WFFs, such as recent work conducted in collaboration with BC and Alberta Wildfire Services (12, 31, 36, 135, 136). However, the comprehensive assessment of chronic cardiorespiratory health and the complementary mechanisms in a Canadian WFF workforce remains limited and presents a major novelty of the CREWS Study.

#### Open enrollment

The CREWS Study has an open enrollment throughout the 3-year timeline allowing for an increasingly diverse group of WFFs. This would increase sample size and may induce sufficient power to analyze data with consideration to factors relating to firefighting experience, crew type, sex, and ethnicity. The WFFEHE study, for example, identified that majority of their participants were experienced WFFs, which may influence the effect size of various findings (especially if an upper or lower limit of the outcome exists). Although we currently have a diverse array of experience in our participant pool, the open enrollment of the CREWS Study allows for increased diversity of experience, likely including more smoke naïve (novice) WFFs to be considered to determine effects of occupational exposure from a true baseline. This will also inform how the health effects of cumulative exposure may differ from initial contact.

### Study challenges

Although an open enrollment will attempt to increase variability across our sample, the cohort is limited to two crew types from a single fire zone. Unit crews are comprised of approximately 20 WFFs, who work on larger, prolonged incidents requiring increased resources, often remaining at a single incident for an entire 14-day deployment (13). The initial attack crews, comprised of 3–4 individuals, are the first response to nascent fires and often remain on standby in their geographic zone, and drive, hike, or fly into smaller and more remote fires with the goal of suppression before the fire turns into a larger incident that requires additional resources (137). The daily operational differences of unit and initial attack crews may influence divergent exposures and health outcomes. However, previously published data that details task-specific exposure, enables the data we collect to be interpreted based on, at the very least, task engagement, which tends to be ubiquitous across diverse crew types (10). Despite enrollment limited to two fire bases within a single BCWS Fire Center, the regular deployment of most crew members to different zones throughout the season enhances the variability to diverse biogeoclimatic zones and fuel sources.

Field-research is fraught with uncontrollable variables which are further exacerbated by the wildland firefighting environment. The interruption of WFFs' occupational duties may risk human life, property and resources. Thus, conducting field research with WFFs requires methodology which is conducive to the field environment and limited to short testing windows with minimal interference to occupational tasks. As such, acute studies in the CREWS Study focus on measures which can be easily applied in the field but may limit the depth of interrogation of specific research questions. Moreover, variables that are typically well-controlled in a laboratory setting such as temperature, humidity, wind, and noise, cannot be controlled for in this field setting. Participant standardizations (food, exercise, nicotine and caffeine cessation) are also limited in the field. Thus, the CREWS Study strives to note relevant field observations, when possible, to provide transparency of such variables in future publications.

Despite efforts to increase equity and diversity in wildland firefighting, according to a 2016 report, only 24.7% of seasonal BCWS employees are women, although this number can vary regionally (138). In the CREWS Study, only 5 of the 56 (~9%) enrolled participants self-reported their biological sex as female, likely owing to the male-skewed sex-distribution of the initial recruitment pool. Some epidemiological evidence suggests that women are more susceptible to the health risks associated with ambient air pollution (139, 140). Moreover, the influence of sex hormones (e.g., estrogen, progesterone, testosterone), which vary greatly across the menstrual cycle in females, can affect inflammation and play a role in endothelial function (141). Although we accounted for self-reported biological sex, menstrual cycle phase, and hormonal contraceptive use, we did not confirm biological sex, nor measure circulating concentration of hormones which, even within a cycle, exhibit great inter-individual heterogeneity (142, 143). Unfortunately, with a limited sample size of enrolled females, we are currently underpowered to make statistical comparisons regarding sex in the present study. However, the open enrollment of the study attempts to ameliorate this disparity across the 3-years. Follow up studies are urgently needed to identify differences in acute and chronic health outcomes and contributing mechanisms in female and intersex WFFs. This likely requires intentional and national collaborative recruitment efforts targeted toward these groups rather than convenience-based sampling.

The research team directly involved in the CREWS Study is relatively small and the mobilization required for midseason testing is rapid, thereby limiting the capacity of the research team to collect data on every deployment from each crew. As such, it will not be possible to directly quantify total and mean exposure of PM_4_, respiratory crystalline silica, and CO for each deployment and/or season. However, the exposure data collected will provide information across acute study periods and will generally add to existing literature regarding task-specific exposure.

The BCWS has provided all operational staff, for optional use, RPE during the 2024 fire season (144) and intends to continue doing so in coming seasons. Three different types of NIOSH-certified respiratory protection, including the 3M Aura 9211+ N95 facepiece filtering respirator, the 3M Quick Latch elastomeric half face respirator, and the 3M Secure Click elastomeric half face respiratory were provided by BCWS as options. Both elastomeric respirators are equipped with a Multi-gas/P100 cartridge. RPE use would reduce participant exposure to PM_4_, inclusive of RCS, therefore affecting cardiorespiratory findings. The CREWS Study accounts for RPE-use during acute studies via direct observation or daily participant recall and relies on more long-term participant recall through questionnaires for the longitudinal study. Thus, interpretation of these outcomes relies on accuracy of the self-reported data and may be over- or underestimated, presenting a study limitation.

### Implications

The aims of the CREWS Study strategically underlies the mission of the ministry responsible for workplace health and safety in BC (WorkSafe BC), which is to prevent workplace injury, illness, disease, and death. The data generated from this study also aligns with the priorities of the BCWS R&I to promote research to enhance health, wellness, and safety within the organization as outlined in their 2022–2025 Research and Innovation Strategic Roadmap (145). The findings generated from the CREWS Study will provide both groups evidence-based guidance to address BCWS's safe work standard on staff health management [objective 1.1 (145)] and may support future intervention strategies [objective 1.2 (145)]. For example, RPE and hygiene intervention protocols, such as those previously outlined by Cherry and colleagues, may become increasingly critical with cumulative exposures explored in the CREWS Study (12, 135). The concurrent exploration of inflammatory mechanisms with cardiorespiratory outcomes may also guide future research initiatives that target these pathways via pharmaceutical [e.g., vitamin C infusion (146)], nutrition [e.g., foods high in anti-inflammatory -oxidants (147), daily prebiotic and/or probiotic supplements (148)], or exercise [e.g., anti-inflammatory exercise bouts (149)] strategies to mitigate health risk in BCWS workers. Finally, the study supports the BCWS shift to total worker health [objective 1.3 (145)] by comprehensively addressing both acute and chronic health outcomes from the cellular to organismal level.

For enrolled participants, although the CREWS Study is not intended to be diagnostic, annual results packets may serve as a personal health monitoring system. This powerful personal health resource allows for research-driven autonomy guiding daily decision-making. This data could inspire changes in risk perception, tolerance, and behaviors thereby impacting the practice of refusing unsafe work, application of mitigation strategies (e.g., RPE use), or even tailoring career length. These findings may also influence hazard pay, union measures, and other legislative pursuits by WFFs and relevant worker organizations. Overall, the data provides more information for WFFs to make personal decisions regarding how to navigate their careers while facing potential health risks.

The high exposure to smoke, dust, and ash experienced by WFFs may not be a problem siloed to the occupation. Indeed, other outdoor worker populations are becoming increasingly susceptible to wildfire-related exposures, a key concern outlined in a recent NIOSH hazard review (107, 111). Additionally, the public, especially groups disproportionately affected by environmental disasters (e.g., rural and Indigenous communities, individuals experiencing homelessness, and low socioeconomic status groups), are also experiencing a growing impact of wildfire activity on their health (150). Understanding how wildfire exposure effects the cardiorespiratory system in WFFs, allows the rapid and targeted expansion of similar research aims to better understand health effects in other vulnerable cohorts, which are needed to create and apply relevant guidelines for health risk management across diverse groups.

The financial burdens of fire are exorbitant and only expected to increase as wildfire seasons become more severe (151). A significant portion of this burden stems from the acute and chronic public healthcare needs associated with extreme wildfire events (152–154). Several examples of economic implications from the CREWS Study include: 1) guidelines and recommendations to reduce public and vulnerable population health risk, 2) production of technologies that target mechanisms to attenuate cardiorespiratory effects, 3) applying subclinical measures and biomarkers for early disease detection and prevention, lessening long term health consequences, 4) allocation of healthcare resources based on chronic health outcomes. While the findings from the CREWS Study will not attenuate the growing incidence of extreme wildfire events, it can prepare our population and its systems to be resilient in the face of climate change.

## Conclusions

In conclusion, the CREWS Study is an ongoing collaborative effort between the BC Wildfire Service and researchers at the University of British Columbia Okanagan, conducted to better understand the chronic cardiorespiratory health effects of wildfire suppression on its workers. In 2024, we enrolled 56 participants across several BCWS unit and initial attack crews to participate in a 3-year repeated measures study examining various measures related to vascular and airway health. This protocol also aims to better understand the acute effects of wildfire exposure that may contribute to the cumulative health effects of consecutive seasons of wildland firefighting. The findings from the CREWS Study form the basis of climate change resiliency by influencing guidelines and recommendations, personal decision-making, mitigation strategy development, and economic factors.

## Author's note

The authors designed and executed the study and have sole responsibility for the writing and content of the manuscript. The content of this work reflects the perspectives and opinions of the authors and does not necessarily reflect the opinions or policy of BCWS.

## Data Availability

The raw data supporting the conclusions of this article will be made available by the authors, without undue reservation.
