# Supplementary material for: CardioRespiratory Effects of Wildfire Suppression (CREWS) study: an experimental overview
Source: Front Public Health. 2025 May 16;13:1578582. doi: 10.3389/fpubh.2025.1578582 (PMC12123879; doi:10.3389/fpubh.2025.1578582)
Supplement: Supplementary file 1 [file Table_1.docx]

**Supplementary Table 1.** Materials and equipment details for the CREWS Study

| **Measurement Name** | **General Equipment** | **Equipment Details** | **Analysis software** | **Protocol Reference** |
| --- | --- | --- | --- | --- |
| **Ongoing Assessments** | | | | |
| Field instruments | Portable cooler (-20°C) | CF55, Alpicool, Guangdong, China | _ | _ |
|  | Portable battery (1260Wh 50.4V) | EF3 Pro, EcoFlow, Shenzhen, China | _ | _ |
| Weight | Digital scale | Taylor USA, Oak Brook, IL | Direct reading | _ |
| Blood pressure | Automated sphygmomanometer | Omron 708-BT, Omron, Japan | Direct reading | _ |
| Questionnaire Data | Survey software | Qualtrics, Provo, UT | _ | _ |
| Spirometry | Handheld, portable spirometer | Easy One Air Spirometry System, ndd, Zurich, Switzerland | Direct reading | (Graham *et al.*, 2019) |
| Impulse Oscillometry | Impulse Oscillometer | Tremoflo C-100 Airwave Oscillometry System, Thorasys, Montreal, Quebec, Canada | Direct reading | (King *et al.*, 2020) |
| Pulmonary Gas Exchange | Non-invasive Gas Exchange Monitor | MediPines Gas Exchange Monitor; MediPines Corp., Yorba Linda, CA | Direct reading | _ |
| Flow Mediated Dilation | High-resolution duplex ultrasound | Terason uSmart 3300, Teratech | FMD/BloodFlow Software version 5.1, Reed C, Australia | (Thijssen *et al.*, 2019) |
| Pulse Wave Velocity | Hand-held tonometer | SPT-301; Millar Instruments, Houston, TX, USA | LabChart v. 8.1; ADInstruments | (Spronck *et al.*, 2024) |
|  | lead-III echocardiogram | FE 132; ADInstruments, Colorado Springs, CO, USA | LabChart v. 8.1; ADInstruments | (Spronck *et al.*, 2024) |
| Sputum Induction | Ultrasonic nebulizer | Universal III, Flaem Medical Devices, Italy | _ | _ |
| Respirable crystalline silica and particulate matter (PM_4_) | Air sampling pump calibrator | DryCal Defender 510, Mesa Labs | _ | _ |
|  | Personal air sampling pumps | GilAir Plus, Sensidyne Ltd. | Assessed via American Industrial Hygienists Association-accredited industrial hygiene laboratory (EMSL Analytical Inc., Mississauga, ON) | NIOSH0600  NIOSH7500  (The NIOSH Manual of Analytical Methods, 2022) |
| Carbon Monoxide | carbon monoxide detector | Tango TX-1, Industrial Scientific | Direct reading | NIOSH 6604  (The NIOSH Manual of Analytical Methods, 2022) |
| **Forthcoming Assessments** | | | | |
| Fractional exhaled nitric oxide (FeNO) | Portable handheld FeNO device | NIOX Vero, Circassia, AB | Direct reading | (Dweik *et al.*, 2011) |
| Heart Rate Variability | lead-III echocardiogram | FE 132; ADInstruments, Colorado Springs, CO, USA | LabChart v. 8.1; ADInstruments | (Heart Rate Variability, 1996) |
| Carboxyhemoglobin saturation (SpCO) | Pulse carbon monoxide oximeter | Rainbow Pulse-CO oximetry; Masimo, Irvine CA, USA | Direct reading | _ |

REFERENCES

Dweik RA, Boggs PB, Erzurum SC, et al. (2011) An Official ATS Clinical Practice Guideline: Interpretation of Exhaled Nitric Oxide Levels (FeNO) for Clinical Applications. *Am J Respir Crit Care Med*; **184**: 602–15. American Thoracic Society - AJRCCM. p. 602–15.

Graham BL, Steenbruggen I, Miller MR, et al. (2019) Standardization of Spirometry 2019 Update. An Official American Thoracic Society and European Respiratory Society Technical Statement. *Am J Respir Crit Care Med*; **200**: e70–88. p. e70–88.

Heart Rate Variability. (1996) . Available at https://www.ahajournals.org/doi/epub/10.1161/01.CIR.93.5.1043. Accessed 12 April 2025.

King GG, Bates J, Berger KI, et al. (2020) Technical standards for respiratory oscillometry. *European Respiratory Journal*; **55**. European Respiratory Society.

Spronck B, Terentes-Printzios D, Avolio AP, et al. (2024) 2024 Recommendations for Validation of Noninvasive Arterial Pulse Wave Velocity Measurement Devices. *Hypertension*; **81**: 183–92. American Heart Association. p. 183–92.

The NIOSH Manual of Analytical Methods. (2022) . Available at https://www.cdc.gov/niosh/nmam/5th_edition_web_book.html. Accessed 21 November 2024.

Thijssen DHJ, Bruno RM, van Mil ACCM, et al. (2019) Expert consensus and evidence-based recommendations for the assessment of flow-mediated dilation in humans. *Eur Heart J*; **40**: 2534–47. p. 2534–47.
