# Supplementary material for: CardioRespiratory Effects of Wildfire Suppression (CREWS) study: an experimental overview
Source: Front Public Health. 2025 May 16;13:1578582. doi: 10.3389/fpubh.2025.1578582 (PMC12123879; doi:10.3389/fpubh.2025.1578582)
Supplement: Supplementary file 2 [file Data_Sheet_1.docx]

CardioRespiratory Effects of Wildfire Suppression (CREWS) Study: An experimental overview

L. Madden Brewster^1^, Drew Lichty^2^, Natasha Broznitsky^3^, Philip N. Ainslie^1^

*^1^Centre for Heart, Lung and Vascular Health, School of Health and Exercise Sciences, University of British Columbia, Kelowna, BC, Canada*

*^2^Canada Wildfire, University of Alberta, Edmonton, AB, Canada*

*^3^Research and Innovation Business Area, British Columbia Wildfire Service, Victoria, BC, Canada*

**Running title:** CardioRespiratory Effects of Wildfire Suppression Study

**Key words:** cardiorespiratory, wildfire, occupational & environmental exposure, occupational health, wildland firefighters

Corresponding Author:

L. Madden Brewster, PhD

University of British Columbia Okanagan

School of Health and Exercise Sciences

3333 University Way

Kelowna, BC V1V 1V7, Canada

Email: [madden.brewster@ubc.ca](mailto:madden.brewster@ubc.ca)

**Supplementary Materials 1.** Detailed Methods

*Anthropometric Variables*

Anthropometric variables were collected at the pre- and post-season timepoints. Height was measured in centimeters using a tape measure fixed to a wall. Weight was collected in kilograms using a digital scale (Taylor USA, Oak Brook, IL). BMI was calculated as: $\frac{Weight (kg)}{{Height (m)}^{2}}$ . Waist circumference was taken at the midpoint between the iliac crest and the last palpable rib, at the end of a normal exhale and measured in millimeters. Blood pressure was measured with an automated blood pressure reader (Omron 708-BT, Omron, Japan) after a minimum of 10 minutes supine. Blood pressure measurements were performed in duplicate unless a difference >3mmHg of either systolic (SBP) or diastolic (DBP) blood pressure between readings was noted. Mean arterial pressure (MAP) was calculated as:

$$MAP (mmHg)= \frac{2*DBP \left( mmHg \right)+SBP\left( mmHg \right)}{3}$$

*Measures of respiratory function*

*Spirometry*: Spirometry was performed according to the 2019 American Thoracic Society and European Respiratory Society’s guidelines to obtain clinical indices of pulmonary function (e.g., forced expiratory volume in 1 second, FEV_1_; forced vital capacity, FVC; FEV_1_/FVC, mid-expiratory flow; peak expiratory flow; forced expiratory flow at 50%) (Graham *et al.*, 2019). Participants were seated upright and instructed how to perform the maneuver using a validated, handheld spirometer (Easy One Air Spirometry System, ndd, Zurich, Switzerland). Briefly, participants were asked to inhale completely, followed by quick, maximal expiration. Participants were encouraged to continue exhaling until a plateau in flow was reached or to volitional maximum exhale, at which point participants were instructed to take a maximal inhale to complete the maneuver. A minimum of three trials were taken for average measurements across the trials unless the device prompted additional trials. Coefficient of variation for the Easy One Air Spirometry System has been shown to be 3.3% for FVC, 2.6% for FEV1, and 1.9% for FEV_1_/FVC with a slight underestimation of these values compared with reference values (Barr *et al.*, 2008).

*Oscillometry*: Impulse oscillometry was used to assess total and peripheral airway impedance via the measurement of airway resistance and reactance. Impulse oscillometry measurements were conducted in accordance with current technical standards outlined by the European Respiratory Society (King *et al.*, 2020) concurrent with manufacturer’s instructions (Tremoflo C-100 Airwave Oscillometry System, Thorasys, Montreal, Quebec, Canada) prior to spirometry and sputum induction protocols. Participants were instructed to sit upright in a chair or back of a vehicle (see Figure 3), feet flat on the ground, with hands supporting their cheeks, noseclip on, and mouth sealed around the mouthpiece. The device was held so that the participant faced approximately 15° upward from the horizontal plane to avoid compression of the upper airways. The participant completed three normal tidal breaths prior to beginning the test at which point, gentle vibrations (frequencies) persisted for 20-seconds while the participant continued normal tidal breathing. A minimum of three trials were conducted, ensuring a coefficient of variation of <10% between trials in accordance with ERS guidelines (King *et al.*, 2020). Trials were excluded based on software recommendation indicating reduced validity (<70%) due to limited breaths across the testing period, low coherence <0.7, or clear interruptions within the test caused by swallowing, coughing, laughing, talking, or leakage of airflow. Reference values used in the interpretation of predictive resistance values (z-scores) were based on Oostveen and colleagues (Oostveen *et al.*, 2013).

*Pulmonary gas exchange*: A validated noninvasive pulmonary gas exchange monitor (MediPines Gas Exchange Monitor; MediPines Corp., Yorba Linda, CA) was used to collect heart rate (HR; from pulse oximetry), peripheral oxygen saturation (SpO_2_), and estimated arterial partial pressure of oxygen (P_a_O_2_) (West and Prisk, 2018). The device measures the partial pressure of end-tidal gasses (oxygen and carbon dioxide) and peripheral oxygen saturation (SpO_2_) during 1-2 minutes of stable tidal breathing. The P_a_O_2_ is estimated from SpO_2_ by correcting for the Bohr effect using end-tidal partial pressure of carbon dioxide. End-tidal partial pressure of oxygen is assumed to be the alveolar partial pressure of oxygen (P_A_O_2_). An ‘oxygen deficit’ is calculated as the difference between P_A_O_2_ and P_a_O_2_. This assessment of gas exchange – similar to the alveolar to arterial O_2_ gradient – has been validated across healthy and patient populations (West *et al.*, 2019; Howe *et al.*, 2020).

*Measures of vascular function*

*Flow mediated dilation:* Reactive hyperemia flow mediate dilation (RH-FMD) of the brachial artery was used to assess endothelium-dependent vasodilatory function according to international guidelines (Thijssen *et al.*, 2011). A high-resolution duplex ultrasound (Terason uSmart 3300, Teratech) with a 10-MHz multifrequency linear array probe (15L4 Smart Mark, Teratech) was used to simultaneously measure diameter and blood velocity (insonation angle, 60°) of the distal third of the left brachial artery. Ultrasound landmarks were used to ensure that the same segment of the artery was imaged for each measurement and that gain and dynamic range were maintained. Screen capture of the ultrasound measurements were saved as audio-video interleave file (Camtasia Studio) for future analysis by use of edge-detection software (Woodman *et al.*, 2001).

Edge detection software (FMD/BloodFlow Software version 5.1, Reed C, Australia) was used to analyze the RH-FMD. Briefly, a region of interest was selected around the highest quality portion of the B-mode longitudinal arterial image and another around the Doppler blood velocity trace. Automatic and continuous tracking of the vessel walls and velocity trace within the regions of interest at a frequency of 30 Hz was performed by the software. A moving window-smoothing function (smoothed median across time) was used to automatically detect peak diameter post-cuff deflation. The relative (%) and absolute (mm) difference between peak and baseline diameters was RH-FMD. The shear stress area under the curve (SS_AUC60_) 60-seconds after deflation of the cuff was considered the stimulus for RH-FMD (Pyke, Dwyer, and Tschakovsky, 2004; Pyke and Tschakovsky, 2007). Indices of resistance vessel function such as peak and total reactive hyperemia were obtained (Limberg *et al.*, 2020; Rosenberry and Nelson, 2020). Peak reactive hyperemia was calculated as the greatest 3-second post-deflation blood flow whereas total reactive hyperemia was the blood flow area under the curve (AUC) 3-minutes after the cuff was deflated. The average coefficient of variation for FMD within the same individual between sessions by our group is 10.3%.

*Pulse wave velocity:* The PWV between the carotid and femoral artery is the gold standard for the noninvasive assessment of central arterial stiffness and was conducted according to current expert consensus (Spronck *et al.*, 2024). A hand-held tonometer (SPT-301; Millar Instruments, Houston, TX, USA) was used to sequentially measure pressure at the carotid and femoral sites concurrent to a lead-III echocardiogram (FE 132; ADInstruments, Colorado Springs, CO, USA). Instruments were connected to either a bridge (tonometer; Bridge Amp/FE221, ADInstruments, Colorado Springs, CO, USA) or bio amplifier (ECG; Dual Bio Amp/FE232, ADInstruments, Colorado Springs, CO, USA) and were integrated with an analog-to-digital converter (Powerlab/16SP ML 880; ADInstruments, Colorado Springs, CO, USA) to a laptop interface with data acquisition software (LabChart v. 8.1; ADInstruments) to capture concurrent ECG and tonometry signals. A secondary channel calculating heart rate was used to ensure there was not a significant (>5%) change in heart rate between sequential measurements. A minimum of 60-seconds of tonometry data was recorded at each site. The distance from each measurement site to the supra-sternal notch was noted to the nearest 0.5 centimeter.

Pulse transit times were averaged over at least 20 cardiac cycles for each measurement site. The time between the R-wave and the inflection of the pressure wave was determined as the ECG-carotid and ECG-femoral pulse transit time. Central pulse transit time (PTT) was calculated as the difference between femoral and carotid PTT:

$Central PTT \left( s \right)=Femoral PTT \left( s \right)-Carotid PTT \left( s \right)$*.*

Central pulse wave velocity was determined by the following equation:

$$PWV \left( \frac{m}{s} \right)=\frac{Distance (m)}{Central PTT (s)}$$

*Circulating and airway-specific inflammation*

Venous blood was collected and processed for future exploratory analysis of established circulating markers involved in vascular (Ballou and Lozanski, 1992; Adams and Shaw, 1994; Sun *et al.*, 2012) and pulmonary (Hermans *et al.*, 2001; Corbel *et al.*, 2002; Eisner *et al.*, 2003; Papiris *et al.*, 2018; Singh, Anshita, and Ravichandiran, 2021) inflammatory pathways and injury.

Whole blood from an arm vein was drawn by a trained research technician. Briefly, a 23-gauge butterfly needle connected to an access device was used to collect venous whole blood into tubes containing either clot activators and serum gel separator (SST) or ethylenediaminetetraacetic acid (EDTA) for serum or plasma isolation respectively. After collection, SST tubes were set upright to coagulate for 30-minutes followed by centrifugation at 3800 RPM (2009 RCF) at room temperature. EDTA tubes were centrifuged immediately at 3300 RPM (1514 RCF) for 10 minutes at room temperature. Plasma and serum were aliquoted and snap frozen in liquid nitrogen (-196°C) for up to 14-days before transferring samples to -80°C freezer for long-term storage.

Sputum samples were collected for the analysis of relevant markers of inflammatory cell differential (see table 2) (Frigas *et al.*, 1981; Brown *et al.*, 1987; Dent *et al.*, 2004; Al Obaidi *et al.*, 2009; Zhu *et al.*, 2014). A 230V~50Hz ultrasonic nebulizer (Universal III, Flaem Medical Devices, Italy) was used to vaporize hypertonic (7%) saline for inhalation to induce sputum production. Participants were instructed to inhale the vapor for three 7-minute periods while wearing a noseclip. Between each round, participants rinsed their mouth with water and blew their nose and were encouraged to wheeze and belly-heave to induce sputum production from the deep airways. When participants produced phlegm clearly associated with the upper airways (post-nasal drip and throat-scraping cough), participants were asked to spit these products into a waste cup. Once the participant confirmed they could not produce anymore sputum, they then completed a spirometry test to ensure that their baseline FEV_1_ had not dropped more than 20%. Contingent upon within-range FEV_1_ measures, participants continued up to 3 rounds of nebulized hypertonic saline inhalation followed by sputum induction. Sputum was stored at -20°C for up to 4-weeks before being processed for supernatant components.

Sputum samples were thawed, and the plugs were separated and weighed. Sputum plugs were washed in phosphate buffered saline and centrifuged at 500 RCF for 10 minutes at 4°C. A portion of the untreated supernatant was collected and stored at -80°C for future analysis. The remaining sample was treated with Diothiothreitol SPUTOLYSIN (Millipore Sigma, #560000), diluted with phosphate buffered saline, filtered (40um mesh strainer), and centrifuged at 500 RCF for 10 minutes at 4C. The Diothiothreitol-treated supernatant was collected and stored at -80°C for future analysis. Multiplex (e.g., Bio-Plex Multiplexing, Mesoscale Discovery) and enzyme-linked immunoassays will be used to assess samples for serum and sputum markers described above.

*Exposure assessments*

*Toenail samples:* Human toenail samples were collected pre- and post-season for the analysis of toxic metal exposure by inductively coupled plasma mass spectrometry (ICP-MS) (Salcedo-Bellido *et al.*, 2021). To prepare toenails for analysis, the toenail is first washed and processed via microwave assisted acid digestion, followed by the measurement of specific trace metals by ICP-MS (Button *et al.*, 2009).

*Air monitoring:* For each midseason sampling day, 2-4 WFFs per day were selected for air monitoring, which involved wearing a personal sampling pump (GilAir Plus, Sensidyne Ltd.) and carbon monoxide detector (Tango TX-1, Industrial Scientific). The personal air pump collected respirable particulate (with an aerodynamic diameter cut-point of 4 µm) via NIOSH 0600 and respirable crystalline silica, as alpha quartz, via NIOSH 7500, while carbon monoxide was assessed according to NIOSH 6604 (The NIOSH Manual of Analytical Methods, 2022). Briefly, the air sampling pumps were connected via quarter inch tubing to a 37-mm three-piece cassette PVC filter with a pore size of 5 microns. A conductive nylon cyclone operating at 1.7 litres per minute was used as a sampling inlet to achieve a respirable curve with a median 50% cut-point of 4 microns, in accordance with American Conference of Governmental Industrial Hygienists (ACGIH) and International Organization for Standardization (Air quality — Particle size fraction definitions for health-related sampling, 1995; The NIOSH Manual of Analytical Methods, 2022). The sampling pumps were calibrated pre- and post-shift using a DryCal Defender 510 (Mesa Labs). The carbon monoxide detectors were zeroed and calibrated prior to each sampling trip according to the manufacturer’s instructions, using a 100 ppm CO cylinder. The sampling inlet and the carbon monoxide detectors were placed as close to the breathing zone as possible, often clipped to the lapel of the Nomex shirt or the straps of a chest-pack. Field blanks were collected each sample day and submitted to the lab for analysis along with their respective samples.

All filters were analyzed by an American Industrial Hygienists Association-accredited industrial hygiene laboratory (EMSL Analytical Inc., Mississauga, ON). The filters were purchased pre-weighed and were post-weighed by the lab using a mass balance with a sensitivity of 0.001 mg. Crystalline silica, as quartz, was analyzed via x-ray diffraction. Both PM_4_ and silica are reported by mass concentration (mg/m^3^ and µg/m^3^, respectively) according to the following equation:

$$Mass Concentration \left( \frac{mg}{m^{3}} \right)=\frac{\left( Filter Post Weight \left( mg \right)-Filter Pre Weight \left( mg \right) \right)}{\left( Flow rate \left( \frac{m^{3}}{min} \right)*Duration \left( min \right) \right)}$$

All air sampling began at the staging area, just prior to engaging on the fire line, and ended back at the staging area when participants had exited from the fire line. Thus, samples represent a fire line average and not a shift-length average.

The research team accompanied WFF crews from the staging area to the fire line to ensure that air monitoring equipment was functioning properly and to collect field observations regarding environmental characteristics, fire behavior, RPE use, and task engagement. The research team attempted to follow crew members wearing monitoring equipment throughout their shift. Individual WFFs were sometimes assigned to different areas of the fire. As a result, the research team was not always able to keep continuous records on everyone, and instead relied on regular (every 1-3 hours) check-ins and subject recall for daily task engagement and RPE use.

REFERENCES

Adams DH, Shaw S. (1994) Leucocyte-endothelial interactions and regulation of leucocyte migration. *The Lancet*; **343**: 831–6. Elsevier. p. 831–6.

Air quality — Particle size fraction definitions for health-related sampling. (1995) . Available at https://www.iso.org/obp/ui/en/#iso:std:iso:7708:ed-1:v1:en. Accessed 10 January 2025.

Al Obaidi AHA, Al Samarai AGM, Al-Janabi J, Yahia A. (2009) The Predictive Value of Eosinophil Cationic Protein and Lactate Dehydrogenase in Asthma: A Comparative Study of Serum Versus Sputum. *World Allergy Organization Journal*; **2**: 144–9. p. 144–9.

Ballou SP, Lozanski G. (1992) Induction of inflammatory cytokine release from cultured human monocytes by C-reactive protein. *Cytokine*; **4**: 361–8. p. 361–8.

Barr RG, Stemple KJ, Mesia-Vela S, et al. (2008) Reproducibility and Validity of a Handheld Spirometer. *Respir Care*; **53**: 433–41. p. 433–41.

Brown MA, Pierce JH, Watson CJ, Falco J, Ihle JN, Paul WE. (1987) B cell stimulatory factor-1/interleukin-4 mRNA is expressed by normal and transformed mast cells. *Cell*; **50**: 809–18. p. 809–18.

Button M, Jenkin GRT, Harrington CF, Watts MJ. (2009) Human toenails as a biomarker of exposure to elevated environmental arsenic. *J Environ Monit*; **11**: 610–7. The Royal Society of Chemistry. p. 610–7.

Corbel M, Belleguic C, Boichot E, Lagente V. (2002) Involvement of gelatinases (MMP-2 and MMP-9) in the development of airway inflammation and pulmonary fibrosis. *Cell Biol Toxicol*; **18**: 51–61. p. 51–61.

Dent G, Hadjicharalambous C, Yoshikawa T, et al. (2004) Contribution of eotaxin-1 to eosinophil chemotactic activity of moderate and severe asthmatic sputum. *Am J Respir Crit Care Med*; **169**: 1110–7. p. 1110–7.

Eisner MD, Parsons P, Matthay MA, Ware L, Greene K, Acute Respiratory Distress Syndrome Network. (2003) Plasma surfactant protein levels and clinical outcomes in patients with acute lung injury. *Thorax*; **58**: 983–8. p. 983–8.

Frigas E, Loegering DA, Solley GO, Farrow GM, Gleich GJ. (1981) Elevated levels of the eosinophil granule major basic protein in the sputum of patients with bronchial asthma. *Mayo Clin Proc*; **56**: 345–53. p. 345–53.

Graham BL, Steenbruggen I, Miller MR, et al. (2019) Standardization of Spirometry 2019 Update. An Official American Thoracic Society and European Respiratory Society Technical Statement. *Am J Respir Crit Care Med*; **200**: e70–88. p. e70–88.

Hermans C, Petrek M, Kolek V, et al. (2001) Serum Clara cell protein (CC16), a marker of the integrity of the air-blood barrier in sarcoidosis. *Eur Respir J*; **18**: 507–14. p. 507–14.

Howe CA, MacLeod DB, Wainman L, Oliver SJ, Ainslie PN. (2020) Validation of a Noninvasive Assessment of Pulmonary Gas Exchange During Exercise in Hypoxia. *CHEST*; **158**: 1644–50. Elsevier. p. 1644–50.

King GG, Bates J, Berger KI, et al. (2020) Technical standards for respiratory oscillometry. *European Respiratory Journal*; **55**. European Respiratory Society.

Limberg JK, Casey DP, Trinity JD, et al. (2020) Assessment of resistance vessel function in human skeletal muscle: guidelines for experimental design, Doppler ultrasound, and pharmacology. *American Journal of Physiology-Heart and Circulatory Physiology*; **318**: H301–25. American Physiological Society. p. H301–25.

Oostveen E, Boda K, van der Grinten CPM, et al. (2013) Respiratory impedance in healthy subjects: baseline values and bronchodilator response. *Eur Respir J*; **42**: 1513–23. p. 1513–23.

Papiris SA, Tomos IP, Karakatsani A, et al. (2018) High levels of IL-6 and IL-8 characterize early-on idiopathic pulmonary fibrosis acute exacerbations. *Cytokine*; **102**: 168–72. p. 168–72.

Pyke KE, Dwyer EM, Tschakovsky ME. (2004) Impact of controlling shear rate on flow-mediated dilation responses in the brachial artery of humans. *Journal of Applied Physiology*; **97**: 499–508. American Physiological Society. p. 499–508.

Pyke KE, Tschakovsky ME. (2007) Peak vs. total reactive hyperemia: which determines the magnitude of flow-mediated dilation? *Journal of Applied Physiology*; **102**: 1510–9. American Physiological Society. p. 1510–9.

Rosenberry R, Nelson MD. (2020) Reactive hyperemia: a review of methods, mechanisms, and considerations. *Am J Physiol Regul Integr Comp Physiol*; **318**: R605–18. p. R605–18.

Salcedo-Bellido I, Gutiérrez-González E, García-Esquinas E, et al. (2021) Toxic metals in toenails as biomarkers of exposure: A review. *Environ Res*; **197**: 111028. p. 111028.

Singh S, Anshita D, Ravichandiran V. (2021) MCP-1: Function, regulation, and involvement in disease. *Int Immunopharmacol*; **101**: 107598. p. 107598.

Spronck B, Terentes-Printzios D, Avolio AP, et al. (2024) 2024 Recommendations for Validation of Noninvasive Arterial Pulse Wave Velocity Measurement Devices. *Hypertension*; **81**: 183–92. American Heart Association. p. 183–92.

Sun Z, Li X, Massena S, et al. (2012) VEGFR2 induces c-Src signaling and vascular permeability in vivo via the adaptor protein TSAd. *J Exp Med*; **209**: 1363–77. p. 1363–77.

The NIOSH Manual of Analytical Methods. (2022) . Available at https://www.cdc.gov/niosh/nmam/5th_edition_web_book.html. Accessed 21 November 2024.

Thijssen DHJ, Black MA, Pyke KE, et al. (2011) Assessment of flow-mediated dilation in humans: a methodological and physiological guideline. *American Journal of Physiology-Heart and Circulatory Physiology*; **300**: H2–12. American Physiological Society. p. H2–12.

West JB, Prisk GK. (2018) A new method for noninvasive measurement of pulmonary gas exchange using expired gas. *Respir Physiol Neurobiol*; **247**: 112–5. p. 112–5.

West JB, Wang DL, Prisk GK, et al. (2019) Noninvasive measurement of pulmonary gas exchange: comparison with data from arterial blood gases. *American Journal of Physiology-Lung Cellular and Molecular Physiology*; **316**: L114–8. American Physiological Society. p. L114–8.

Woodman RJ, Playford DA, Watts GF, et al. (2001) Improved analysis of brachial artery ultrasound using a novel edge-detection software system. *Journal of Applied Physiology*; **91**: 929–37. American Physiological Society. p. 929–37.

Zhu A, Ge D, Zhang J, et al. (2014) Sputum myeloperoxidase in chronic obstructive pulmonary disease. *Eur J Med Res*; **19**: 12. p. 12.
