# Supplementary material for: CardioRespiratory Effects of Wildfire Suppression (CREWS) study: an experimental overview
Source: Front Public Health. 2025 May 16;13:1578582. doi: 10.3389/fpubh.2025.1578582 (PMC12123879; doi:10.3389/fpubh.2025.1578582)
Supplement: Supplementary file 4 [file Data_Sheet_3.docx]

**Supplementary Materials 3.** Questionnaire provided to participants during the midseason regarding the entire season.

**General Information**

Name: __________________________

Date:

No. days spent at this fire:

Fire #:

**Fireline Activities**

**1. What type of attack best describes your deployments since the start of the season?**

Direct Attack

Parallel Attack

Indirect Attack

Initial Attack

**2. Within your crew what is your role?**

Crew member

Crew Leader

Crew Supervisor

Assistant Crew Supervisor

**3. Years of service as wildland firefighter:**

**4.** **What task have you spent the MOST time engaged in since the start of the season?** (Choose One)

Patrol (Green)

Patrol (Black)

Wetlining

Bucker

Faller

Swamper

Ignitions

Pump Operator

Mop-up

Holding

Pump Operator

Lookout

Line Locator

Digging Guard

Hose Lay

**Other (Specify):**

**5.** **What other tasks have you performed since the start of the season?** (Select All That Apply)

Patrol (Green)

Patrol (Black)

Wetlining

Bucker

Faller

Swamper

Ignitions

Pump Operator

Mop-up

Holding

Pump Operator

Lookout

Line Locator

Digging Guard

Hose Lay

**Other (Specify):**

**Smoke Exposure Information**

**6. How would you rate your TYPICAL SMOKE exposure since the start of the season?**

None  Light  Medium  Heavy  Very Heavy

**7.** **How would you rate your WORST SMOKE exposure since the start of the season?**

None  Light  Medium  Heavy  Very Heavy

**8. Did you experience a SEVERE EPISODE OF SMOKE EXPOSURE since the start of the season? If so please indicate how many times you have experienced such an event.**

A severe episode of smoke exposure is whenever you find yourself in smoke that is so thick that **visibility** of the fire area is significantly impaired and/or the specific smoke exposure incident was associated with **sudden and intense symptoms** of cough, eye/nose/throat irritation, shortness of breath, difficulty breathing, chest pain, headache, nausea, or vomiting.

Number of extreme smoke exposure events since the start of the season:

Any additional info:

**Ash and Dust Exposure Information**

**9. How would you rate your TYPICAL ASH & DUST exposure since the start of the season?**

None  Light  Medium  Heavy  Very Heavy

**10.** **How would you rate your WORST ASH & DUST exposure since the start of the season?**

None  Light  Medium  Heavy  Very Heavy

**Symptoms**

**11. Since the start of the season, have you had any of the following symptoms?**

**Cough:**

Mild  Moderate  Severe  None

**Sore or Irritated Throat:**

Mild  Moderate  Severe  None

**Shortness of Breath:**

Mild  Moderate  Severe  None

**Wheezing:**

Mild  Moderate  Severe  None

**Watery/Itchy/Red Eyes:**

Mild  Moderate  Severe  None

**Headache:**

Mild  Moderate  Severe  None

**Fatigue:**

Mild  Moderate  Severe  None

**Chest Tightness or Pain:**

Mild  Moderate  Severe  None

**Nausea:**

Mild  Moderate  Severe  None

**Vomiting:**

Mild  Moderate  Severe  None

**Runny Nose:**

Mild  Moderate  Severe  None

**Respirator Use**

**13. Did you use a respirator since the start of the season?**

Yes  No

**14. If yes, which model of respirator did you use?**

N95  3M Quick Latch  3M Secure Click

Other (please specify):

**15. Which of the following would best describe your respirator use since the start of the season?** (Choose one)

Never or almost never

Once every few days

Once every other day

Once per day

2-3 times per day

3 -4 times per day

5+ times per day

**16. How long would you typically wear your respirator when you chose to put it on?**
